# Incoherent dual regulation by a SAM-II riboswitch controlling translation at a distance

**DOI:** 10.1080/15476286.2022.2110380

**Published:** 2022-08-11

**Authors:** Robina Scheuer, Theresa Dietz, Jonas Kretz, Lydia Hadjeras, Matthew McIntosh, Elena Evguenieva-Hackenberg

**Affiliations:** a Institute of Microbiology and Molecular Biology, University of Giessen, Giessen, Germany; b Chair of Molecular Infection Biology II, Institute of Molecular Infection Biology (IMIB), University of Würzburg, Würzburg, Germany

**Keywords:** S-adenosyl-methionine, riboswitch, translation initiation region, transcription termination, ribosome standby site, RNA stability, sRNA, *metA*, *Sinorhizobium*, Alphaproteobacteria

## Abstract

In *Sinorhizobium meliloti*, the methionine biosynthesis genes *metA* and *metZ* are preceded by S-adenosyl-L-methionine (SAM) riboswitches of the SAM-II class. Upon SAM binding, structural changes in the *metZ* riboswitch were predicted to cause transcriptional termination, generating the sRNA RZ. By contrast, the *metA* riboswitch was predicted to regulate translation from an AUG1 codon. However, downstream of the *metA* riboswitch, we found a putative Rho-independent terminator and an in-frame AUG2 codon, which may contribute to *metA* regulation. We validated the terminator between AUG1 and AUG2, which generates the sRNA RA1 that is processed to RA2. Under high SAM conditions, the activities of the *metA* and *metZ* promoters and the steady-state levels of the read-through *metA* and *metZ* mRNAs were decreased, while the levels of the RZ and RA2 sRNAs were increased. Under these conditions, the sRNAs and the mRNAs were stabilized. Reporter fusion experiments revealed that the Shine–Dalgarno (SD) sequence in the *metA* riboswitch is required for translation, which, however, starts 74 nucleotides downstream at AUG2, suggesting a novel translation initiation mechanism. Further, the reporter fusion data supported the following model of RNA-based regulation: Upon SAM binding by the riboswitch, the SD sequence is sequestered to downregulate *metA* translation, while the mRNA is stabilized. Thus, the SAM-II riboswitches fulfil incoherent, dual regulation, which probably serves to ensure basal *metA* and *metZ* mRNA levels under high SAM conditions. This probably helps to adapt to changing conditions and maintain SAM homoeostasis.

## Introduction

Riboswitches are *cis*-acting RNA elements, which are mostly located in bacterial 5′-UTRs, specifically bind a metabolite or an ion, and regulate the expression of the downstream genes [1]. The largest group of riboswitches interact with the *S*-adenosyl-L-methionine (SAM), the main methyl donor for biochemical reactions in the cell, and usually regulate genes of the sulphur metabolism [2–6]. Based on structural features, the SAM-responsive riboswitches are currently assigned to six classes [Mirihana 2, 7]. For the SAM-II riboswitch class that is present in many Alphaproteobacteria [8], the SAM binding mechanism is well understood [9], but the effects on gene expression have not been studied yet.

Gene regulation by riboswitches is based on conformational changes upon ligand binding, which often result in the formation of a transcriptional terminator [5,10] or sequestration of a ribosome-binding site (RBS) [9,11]. Downregulation of translation by a riboswitch could be reversible due to binding and release of the ligand when it is faster than the half-life of the regulated mRNA [12]. On the other hand, translation repression by riboswitches can provoke downstream Rho-dependent transcription termination [13]. Furthermore, dual-acting riboswitches were described, which inhibit translation and destabilize the mRNA by exposing an RNase cleavage site upon ligand binding [14]. The latter examples show coherent dual (down-) regulation by riboswitches, which ensures tight control of gene expression.

Riboswitches that activate gene expression upon ligand binding were also described [1,15]. An interesting recent example is a guanidine binding riboswitch in *Legionella pneumophila*, which upregulates translation of a guanidine efflux pump gene and concomitantly stabilizes the mRNA. The mRNA stabilization is mediated by pseudoknot formation in the ligand-bound aptamer, which serves as a roadblock for transcript scanning by the 5′-monophosphate binding endoribonuclease RNase E [16], the major mRNA degrading endoribonuclease in Proteobacteria [17]. In this case, a coherent dual regulation is again observed: Both riboswitch mechanisms serve to upregulate gene expression, thereby cooperating to increase the regulatory effect.

Although many SAM-II aptamers are followed by predicted transcriptional terminators [8], the mechanism of SAM binding is best understood for a compact SAM-II riboswitch from a Sargasso Sea metagenome, which probably regulates translation. The aptamer of this riboswitch is followed by a predicted RBS whose Shine–Dalgarno (SD) sequence is sequestered in a pseudoknot structure upon SAM binding [9]. The Sargasso Sea metagenome riboswitch was extensively analysed by several research groups using biochemical and biophysical methods such as crystallography, NMR, SAXS and atomistic molecular dynamic simulations [9,18–21]. These studies revealed that in the ligand-unbound state, the riboswitch structure is pre-organized to enable fast and specific SAM binding, which then stabilizes the pseudoknot, thereby occluding the SD sequence. Considering the above-mentioned mechanism of mRNA stabilization by a pseudoknot [16], it can be hypothesized that upon SAM binding, the Sargasso Sea metagenome riboswitch stabilizes the mRNA whose translation it downregulates, thus mediating regulation in two opposite directions. Such a ‘paradoxical’ mode of action can provide robustness in biological systems and is named dual incoherent regulation [22,23]. However, the physiological role of this riboswitch and of SAM-II riboswitches in general was not analysed experimentally.

The soil dwelling plant symbiont *Sinorhizobium meliloti* belongs to the Alphaproteobacteria and harbours two SAM-II riboswitches located upstream of the methionine biosynthesis genes *metA* and *metZ* [8]. In this work, we analysed the levels and the stabilities of *metA* and *metZ* mRNAs and corresponding, riboswitch-containing small RNAs (sRNAs) in *S. meliloti* under high and low SAM conditions. Our results suggest that both SAM-II riboswitches exhibit incoherent dual functions, which probably contribute to the cellular SAM homoeostasis. We also used *egfp* reporter fusions to analyse the regulation of *metA* expression in detail. Our results show that *metA* is transcribed from a strong, regulated promoter. However, transcription into the gene is restricted by a transcriptional terminator, which is located downstream of the translation-regulating riboswitch. According to our data, the riboswitch controls the accessibility of an SD sequence, which is important for initiation of translation 74 nucleotides downstream in the read-through mRNA. This is the first indication that a functional SD sequence does not need to be located close to the AUG translation start.

## Results

### Prediction of the roles of the SAM-II riboswitches located upstream of metZ and metA

**Figure 1.**
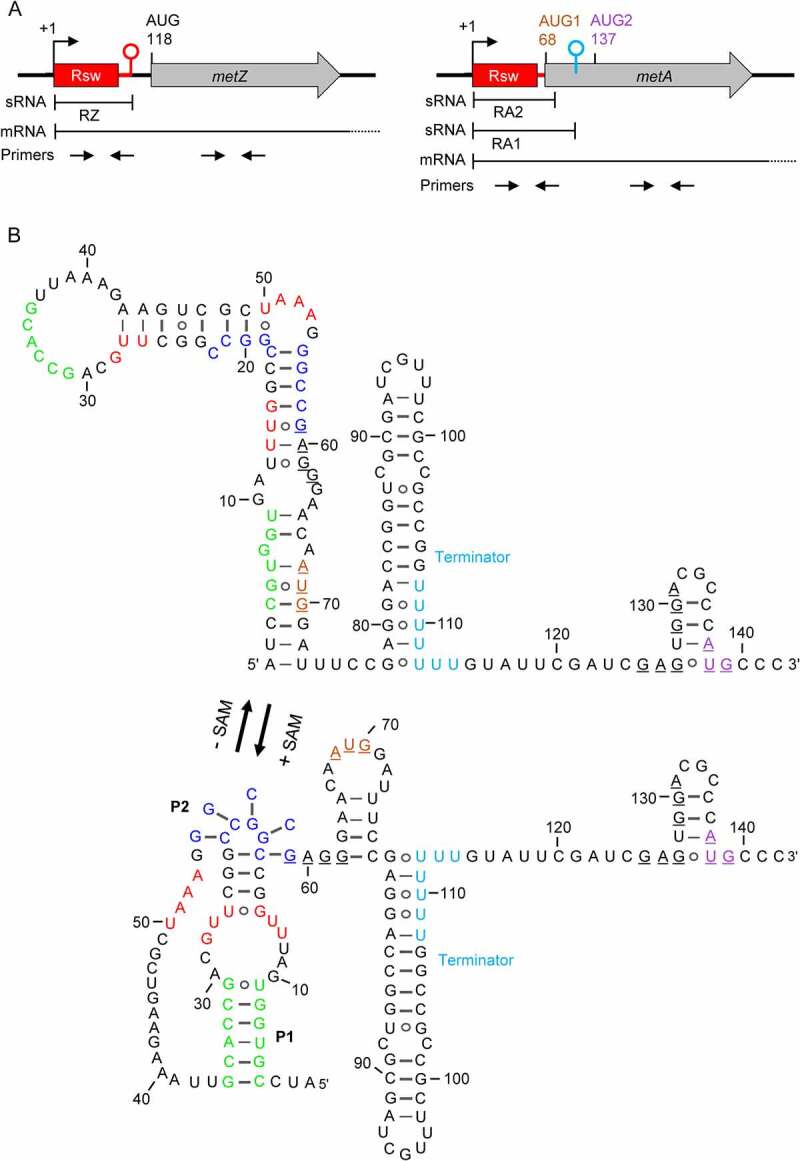
SAM-II riboswitches in *S. meliloti* 2011. A) Scheme of the *metZ* and *metA* genes and their relevant features. The *metZ* and *metA* ORFs are depicted by grey arrows. They are preceded by SAM-II riboswitches depicted in red. Rsw indicates the SAM-binding aptamer. Flexed arrows and hairpins show transcription start sites (TSSs at positions +1) and transcriptional terminators, respectively. Positions of adenine in (putative) AUG start codons are given. Regions corresponding to the riboswitch-containing sRNAs RZ, RA1 and RA2, and to the *metZ* and *metA* mRNAs are indicated (mRNA terminators are not shown). Indicated are also the positions of the primer pairs used for qRT-PCR analysis. B) The *metA* riboswitch and downstream sequences. Shown are the predicted alternative RNA structures adopted without SAM binding (-SAM) and upon interaction with SAM (+ SAM). Red nucleotides: the SAM-binding pocket (SBP); green nucleotides: sequences building stem 1 (P1) upon SAM binding; dark blue nucleotides: sequences building a pseudoknot (P2) upon SAM binding (according to [8]). The SD-like sequence and putative AUG start codons are underlined. AUG1 and AUG2 are coloured according to panel A). The U stretches in the transcriptional terminator between AUG1 and AUG2 are shown in cyan. The *metZ* riboswitch is shown in Fig. S1.

In *S. meliloti*, the *metZ* and *metA* genes are preceded by SAM-II riboswitches (Figure 1A [8];). The predicted changes in the secondary structures of the riboswitches upon SAM binding are shown in Figure 1B and Figure S1. According to Figure S1, the *metZ* riboswitch controls gene transcription, since upon SAM binding by the sensor domain (aptamer), the expression platform adopts a secondary structure of a typical Rho-independent terminator. For the *metA* riboswitch, the prediction suggests that SAM binding probably controls translation, because downstream of the aptamer, a potential ribosome binding site (RBS) is located (Figure 1B). This RBS comprises the four-nucleotide Shine–Dalgarno (SD) sequence GAGG and an AUG (designated AUG1, see Figure 1A). In the SAM-bound state, the P2 pseudoknot structure should interfere with ribosome binding (Figure 1B). However, downstream of AUG1, a potential Rho-independent terminator is located, which is not predicted to be affected by SAM binding to the riboswitch. Moreover, further downstream a second, in-frame AUG is present (designated AUG2), which is preceded by GAG and GGA as potential SD sequences (Figure 1). Thus, the identity of the *metA* start codon and the role of the *metA* riboswitch are not clear.

### Opposite changes in the riboswitch-sRNA and mRNA amounts under high and low SAM conditions

**Figure 2.**
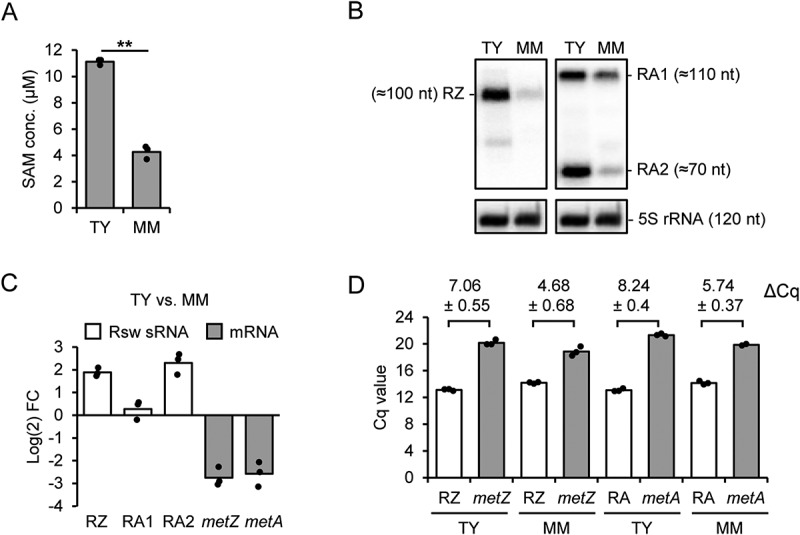
Analysis of SAM levels and RNA steady state amounts in *S. meliloti* 2011 cultures grown in TY and MM. A) SAM concentration in cell lysates. B) Northern blot hybridization of total RNA separated in denaturing 10% polyacrylamide (PAA) gel with probes directed against the *metZ* and *metA* riboswitch aptamer (see Fig. S3). The membrane was first hybridized with the probe detecting the RZ sRNA and then rehybridized with the probe detecting RA1 and RA2 sRNAs (compare to Figure 1A). To determine the sRNA lengths, the membrane was then hybridized sequentially with probes detecting tRNA^Thr^ (86 nt), 6S RNA (156 nt) and 5S rRNA (120 nt). The latter is also shown as loading control. C) Comparison of the sRNA and mRNA levels in TY and MM cultures shown as log_2_ fold change (FC). The sRNA levels were determined by Northern hybridization and the mRNA levels by qRT-PCR analysis. TRIzol-purified total RNA was used. D) Cq values of the qRT-PCR analysis conducted with primers detecting the indicated mRNAs and sRNAs (see Figure 1A; the sRNA-detecting primers target also the 5’-UTR of the corresponding mRNA; RA: RA1 + RA2). Hot phenol-purified total RNA was used. Comparison of results obtained with TRIzol- and hot phenol-purified RNA is shown in Fig. S4. Differences in the Cq values are given (means and s. d.), which indicate much higher abundance of the sRNAs in comparison to the mRNAs. All graphs show means and single data points of three independent experiments.

The SAM-II riboswitches are expected to regulate *metZ* and *metA* expression according to the cellular SAM level. This regulation probably affects the levels of the mRNAs and of the expected riboswitch-containing sRNAs. To address this, we cultivated bacteria under high and low SAM conditions. We anticipated that complex medium (TY) provides high SAM conditions due to the external methionine supply, while in minimal medium (MM), bacteria must synthesize methionine and low SAM conditions prevail. We measured the SAM concentration in crude lysates from *S. meliloti* 2011 cells grown in each medium and confirmed significantly higher SAM levels in the TY-derived lysates (Figure 2A).

Before testing whether the SAM level differences correlate with differences in the levels of RNAs, we asked whether the *S. meliloti* 2011 cultures in TY and MM differ in cell size, count, mass, and in the RNA amount. At OD_600 nm_ = 0.5, the cells in TY were almost twofold larger than in MM (Figure S2A), while the cell count in MM was almost twofold higher than in TY (Figure S2B). Further, there was neither significant difference in the cell mass nor in the RNA amount obtained from identical volumes of TY- or MM-cultures (Figure S2C and Figure S2D). Based on this, in the following we used similar amounts of total RNA to compare sRNA and mRNA levels in cultures grown in the two media (thus, we compared the RNA levels per mass and not per cell).

The levels of the riboswitch-containing sRNAs were analysed by Northern blot hybridization after separation of total RNA in 10% polyacrylamide-urea gel. The radioactively labelled probes were directed against the conserved sensor domains of the riboswitches (Figure S3). Using the *metZ*-directed probe, a signal corresponding to an sRNA with a length of approximately 100 nt was obtained (Figure 2B). This fits to the size of a *metZ* riboswitch sRNA (RZ sRNA) arising by transcriptional termination (see Figure 1A and Figure S1). The level of the RZ sRNA was significantly higher in TY than in MM (Figure 2C). Using the probe directed to the *metA*-riboswitch, two major signals were detected, which correspond to sRNAs with approximate lengths of 110 nt (RA1 sRNA) and 70 nt (RA2 sRNA) (Figure 2B). While the RA1 sRNA level did not differ significantly in the two media, the RA2 sRNA strongly accumulated in TY (Figure 2C). Most probably, the majority of RA1 sRNA arises by transcriptional termination downstream of AUG1 and is processed into the shorter RA2 sRNA. The RA2 accumulation under high SAM conditions suggests that it probably contains the riboswitch in a SAM-bound form.

Since it was not possible to detect the *metZ* and *metA* mRNAs by Northern blot hybridization, we decided to use qRT-PCR for their analysis. Previously, we used *rpoB* mRNA as an internal standard in qRT-PCR [24]. However, the *rpoB* mRNA Cq was typically 22 for the RNA from TY cultures and 20 for the RNA from MM cultures, showing that the level of this mRNA differs in the two media. Therefore, we used a spike-in transcript as a normalization control (see Materials and Methods). The qRT-PCR results revealed significantly lower *metZ* and *metA* mRNA levels in TY when compared to MM cultures (Figure 2C).

To address the relative cellular levels of the sRNAs and the mRNAs, we analysed both RNA classes by qRT-PCR in order to compare their Cq values. The above results were obtained with RNA purified with TRIzol, a method leading to enrichment of sRNAs and low recovery of large RNAs [25,26]. To achieve maximal recovery of all RNA species, we decided to use hot phenol for the extraction and purification of total RNA. The ensuing qRT-PCR analysis was conducted with primers detecting the sRNAs and the 5’-UTRs of the mRNAs or the coding regions of the mRNAs (see Figure 1A above). Figure 2D shows that much lower Cq values were obtained with the sRNA-detecting primers than with the mRNA-specific primers. Since all primer pairs showed similar amplification efficiencies (Table S2), the ΔCq values of 5 to 6 cycles in RNA extracted from MM cultures and 7 to 8 cycles in RNA from TY cultures strongly suggest that under both growth conditions, the sRNAs are much more abundant than the mRNAs. Furthermore, the higher ΔCq values in TY (Figure 2D) are in line with higher sRNA levels and lower mRNA levels under these conditions (see Figure 2C).

The observed downregulation of the *metZ* and *metA* mRNAs with the concomitant upregulation of the RZ and RA2 sRNAs in TY medium is in line with a SAM-dependent post-transcriptional regulation. To validate the SAM-dependence of the RNA levels, we constructed the strain 2011 *FLAG-metK*, in which the expression of the essential SAM synthetase gene *metK* can be monitored and the cellular SAM amount can be manipulated. This mutant strain contains plasmid pKACIT-FLAG-metK integrated in the chromosome. The integration of this plasmid was guided via homologous recombination and resulted in two desired outcomes: the fusion of a triple FLAG-tag motif to *metK* and placing *FLAG-metK* under the control of an IPTG-inducible promoter. The advantage of this approach was that in the absence of IPTG, the expression of *metK* was repressed and therefore the production of SAM was prevented. This modified strain was cultivated in TY medium overnight in the presence of IPTG. From this starter culture, fresh TY medium with various IPTG concentrations was inoculated. Lower concentrations of IPTG corresponded with lower levels of the FLAG-MetK protein and SAM (Figure S5A and S5B). As expected, lower concentrations of IPTG correlated with lower levels of *FLAG-metK* mRNA and an increase in the levels of *metZ* and *metA* mRNAs (Figure S5C). Further, decreasing IPTG concentrations led to decreased levels of the RZ, RA1 and RA2 sRNAs (Figure S5). Together, these results are in line with SAM-dependent i) downregulation of *metZ* and *metA* mRNAs and ii) upregulation of riboswitch-containing sRNAs.

We note that RA2 accumulation was strongly impacted by *FLAG-metK* expression. However, a comparison of the relative RA1 and RA2 levels in WT and the 2011 *FLAG-metK* strain at 0.5 mM and 1 mM IPTG (Figure S5D) suggests that the IPTG-mediated induction of the *FLAG-metK* gene did not perfectly restore the wild-type conditions. Therefore, in the following experiments the parental strain 2011 grown in TY or MM was used.

### Stabilization of the metZ and metA sRNAs and mRNAs under high SAM conditions

**Figure 3.**
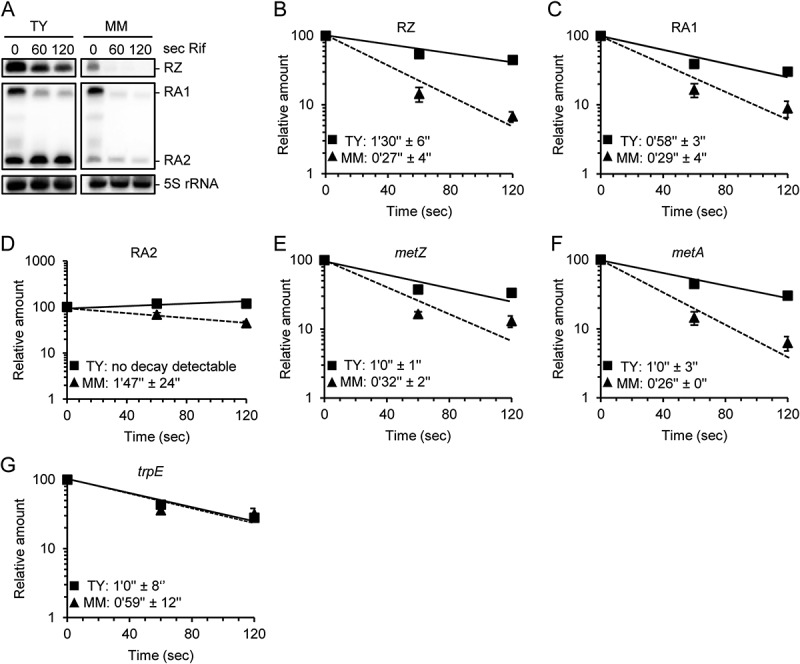
Higher stabilities of *metZ* and *metA* mRNAs and of riboswitch sRNAs in TY than in MM. A) Northern blot hybridization of total RNA separated in denaturing 10% PAA gel. Probes detecting the indicated sRNAs were used. Time (in seconds) after addition of rifampicin (Rif), at which the RNA was isolated, and the used media are indicated. B) to G) Graphs showing the half-lives of the indicated sRNAs and mRNAs in TY and MM. Relative RNA amounts were plotted against the time after Rif addition. The RNA amount at time point 0 was set to 100%. Squares and solid line: RNA from TY cultures. Triangles and dashed line: RNA from MM cultures. All graphs show means and standard deviations (s.d.) of two independent experiments. In some cases, the s.d. is smaller than the symbol.

The steady-state level of a transcript is determined by its transcription and stability. To address the question of whether the above shown differences in the RNA levels are due to changes in RNA stability, half-life determinations were conducted. To *S. meliloti* 2011 cultures grown in TY or MM, rifampicin was added to stop transcription, and the kinetics of RNA decay were analysed by Northern hybridization or qRT-PCR (Figure 3).

In MM, the RZ and RA1 sRNAs were very unstable with half-lives of approximately 30 seconds (Figure 3B and Figure 3C). Since RA2 is most probably a processed form of RA1, it was not possible to correctly determine its half-life after adding rifampicin, but its amount in MM was reduced twice approximately every 1 min 50 sec (Figure 3D). In TY, the half-life of the RZ sRNA was threefold longer and that of RA1 twofold longer than in MM (Figure 3B and Figure 3C). Decay of RA2 could not be detected in TY (Figure 3D), suggesting that it was strongly stabilized. The stabilization of the RZ and RA2 sRNAs is in line with their accumulation in TY medium (compare to Figure 2B and Figure 2C), which in the case of RZ is most probably also boosted by transcriptional termination upon SAM binding (Figure S1). The stabilization of RA1 sRNA without statistically significant accumulation in TY medium (see Figure 2C above) suggests that under these conditions, transcription from the *metA* promoter is probably downregulated.

Interestingly, we observed that the *metZ* and *metA* mRNAs are also stabilized in a rich medium. Their half-lives were approximately twofold longer in TY than in MM (Figure 3E and Figure 3F), although in TY, their steady-state levels were approximately eightfold lower (compare to Figure 2C). These results strongly suggest that *metZ* and *metA* transcription is downregulated in TY. The *metZ* and *metA* mRNA stabilization in TY was specific since the control *trpE* mRNA was found to have similar half-lives in both media (Figure 3G). Thus, the downregulation of *metZ* and *metA* under high SAM conditions is counteracted by increased mRNA stabilities.

### Transcription of metA: promoter regulation and premature termination

**Figure 4.**
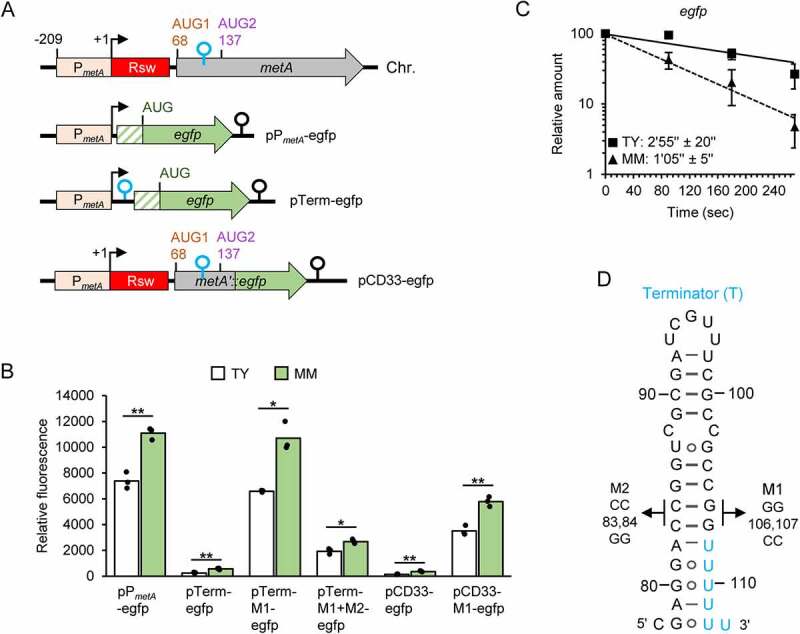
Higher *metA* promoter activity in MM than in TY and transcriptional terminator limiting *metA* expression. A) Schematic representation of *metA* on the chromosome (Chr.) and of used plasmids (indicated) carrying a P*_metA_*-containing region and *egfp* (green arrow). The hatched box represents a synthetic 5’-UTR containing a typical Shine-Dalgarno sequence. Additional elements corresponding to the *metA* transcript are indicated. For other information, see Figure 1A and the text. B) Relative fluorescence of TY and MM cultures of *S. meliloti* 2011 strains carrying the indicated plasmids. The graph shows means of three independent experiments and single measure points representing means from three technical replicates of each independent experiment. C) Half-lives of *egfp* mRNA transcribed from pPmetA-egfp in *S. meliloti* 2011. For details, see Figure 3. Squares and solid line: RNA from TY cultures. Triangles and dashed line: RNA from MM cultures. Shown are means and s.d. of three independent experiments. At some time points of the experiment with TY cultures, the s.d. is smaller than the symbol. D) Predicted secondary structure of the Rho-independent terminator located between AUG1 and AUG2 in the *metA* transcript (compare to Figure 1B). Mutations used in the pTerm-M1-egfp and pTerm-M1+M2-egfp plasmids are indicated.

Our further analysis was focused on *metA*. First, we constructed the promoter fusion plasmid pP_metA_-egfp, in which the *metA* region from −209 to +5 (+1 represents the transcription start site determined previously by others [27]) was fused to a typical bacterial SD sequence followed by *egfp* (Figure 4A). The strain containing this plasmid was cultivated in TY and MM, and fluorescence was measured at OD_600 nm_ = 0.5. The significantly higher fluorescence in MM suggested medium-dependent regulation of the activity of the *metA* promoter (see pP_metA_-egfp in Figure 4B), A result that is in line with the above data (Figure 2C, and Figure 3F). To corroborate this, the reporter gene expression was analysed at the level of RNA. We found that the *egfp* mRNA transcribed from pP_metA_-egfp is less stable in MM than in TY cultures (Figure 4C). Despite this, its steady-state level was higher in MM than in TY (log_2_FC of 0.67 ± 0.2), thus confirming stronger transcription from promoter P*_metA_* during growth in MM. Data obtained using a pP_metZ_-egfp construct support the view that the *metZ* promoter also shows higher activity in MM than in TY cultures (Figure S6).

Next, we tested experimentally the proposed transcriptional terminator between AUG1 and AUG2. When cloned between the strong P*_metA_* promoter and *egfp* on plasmid (Figure 4A), it caused very strong fluorescence reduction (compare pTerm-egfp to pP_metA_-egfp in Figure 4B). M1 mutation in the terminator stem, which is expected to weaken the secondary structure (Figure 4D), led to increased fluorescence (compare pTerm-M1-egfp to pTerm-egfp in Figure 4B), indicating that the transcription termination between P*_metA_* and *egfp* was abolished by the M1 mutation. Introducing a compensatory mutation M2 that should restore the terminator stem (Figure 4D) led to significantly lower fluorescence (compare pTerm-M1+M2-egfp to pTerm-M1-egfp in Figure 4B). The generally higher fluorescence of MM cultures (Figure 4B) can be explained by the stronger activity of P*_metA_* under these conditions. Together, these results confirm the transcriptional terminator shown in Figure 4D.

We also constructed plasmid pCD33-egfp, which contains a translational *egfp* fusion encompassing P*_metA_*, the riboswitch and 33 codons (considering AUG1 as the first codon; Figure 4A). The corresponding fusion transcript harbours AUG1, AUG2 and the transcriptional terminator between them. Cultures containing this plasmid showed very low fluorescence in both TY and MM (Figure 4B). This suggests that despite the strong promoter activity, *metA* expression is quite low in both media (compare pCD33-egfp to pP_metA_-egfp in Figure 4B). Introducing the M1 mutation in pCD33-egfp led to increased fluorescence (compare pCD33-M1-egfp to pCD33-egfp in Figure 4B), demonstrating that the transcriptional terminator between AUG1 and AUG2 strongly limits *metA* expression.

Altogether, our results strongly suggest that *metA* transcription is regulated at the promoter in response to the methionine or SAM availability, and that most transcriptional events are prematurely abolished before AUG2 is transcribed.

### The Shine-Dalgarno sequence in the riboswitch is required for metA translation, which starts at AUG2

**Figure 5.**
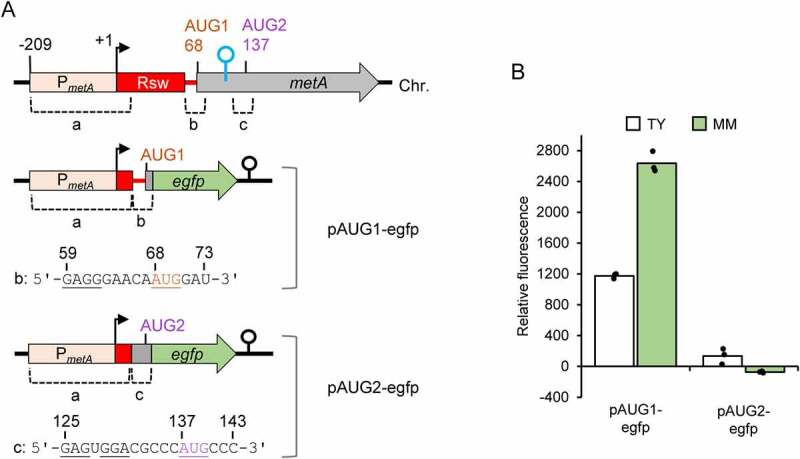
Outside of its native context, the ribosome binding site (RBS) containing AUG1 promotes translation, while the RBS containing AUG2 does not. A) Schematic representation of the translational fusion constructs used for analysis of the two potential RBSs. Chromosomal regions a, b and c, pairwise fused in plasmids pAUG1-egfp and pAUG2-egfp, are indicated. The RNA sequences of the regions b and c are shown and selected nucleotides are numbered (+1 corresponds to the *metA* TSS, see the Chr. scheme). Potential RBS elements (SD and start codons) are underlined. For other details, see Figure 4A and Figure 1A. B) Relative fluorescence of TY and MM cultures of *S. meliloti* 2011 strains carrying the indicated plasmids. The graph shows means of three independent experiments and single measure points representing means from three technical replicates of each independent experiment.

To test whether AUG1 and/or AUG2 can be used as start codon(s) of *metA* in the cell, we constructed two translational fusion plasmids as depicted in Figure 5A. Plasmid pAUG1-egfp corresponds to a transcript that lacks the SAM-II riboswitch aptamer and contains the SD sequence GAGG and AUG1 as a putative RBS for *egfp* translation. Cultures with this plasmid were fluorescent (Figure 5B), suggesting that AUG1 is a start codon. By contrast, cultures containing pAUG2-egfp did not show fluorescence (Figure 5B). The latter plasmid corresponds to a transcript harbouring AUG2 and the upstream SD-like sequences GAG and GGA as a putative RBS (Figure 5A). Our preliminary conclusion was that AUG2 is not the start codon of *metA*.

**Figure 6.**
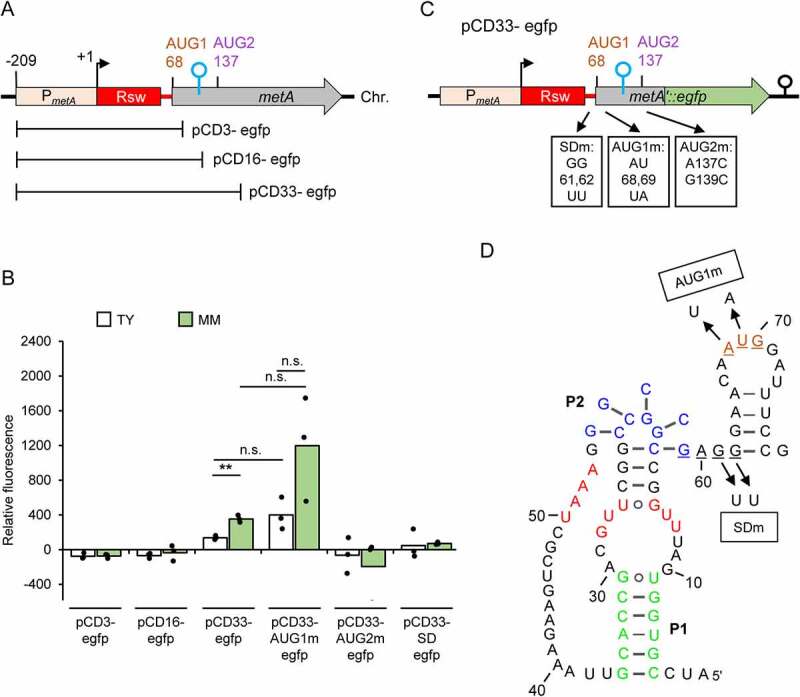
Translation of *metA* starts at AUG2, but requires the SD in the riboswitch. A) Schematic representation of the *metA* regions cloned in the indicated plasmids, which harbour translational *egfp* fusions. B) Relative fluorescence of TY and MM cultures of *S. meliloti* 2011 strains carrying the indicated plasmids. The graph shows means of three independent experiments and single measure points representing means from three technical replicates of each independent experiment; n.s.: not significant. C) Schematic representation of the pCD33-egfp plasmid and the used mutations (indicated at the RNA level). For other details, see Figure 4A and Figure 1A. D) Predicted secondary structure of the riboswitch in its SAM-bound form (compare to Figure 1B). Shown are the mutations used in the pCD33-AUG1m-egfp and pCD33-SDm-egfp plasmids.

Surprisingly, the opposite was observed in translational fusions containing the riboswitch aptamer. We used three additional reporter plasmids, each containing P*_metA_*, the aptamer, and 3, 16 or 33 codons (considering AUG1 as the first codon) fused to *egfp* (Figure 6A). Cultures containing pCD3-egfp or pCD16-egfp (both correspond to transcripts harbouring only AUG1) did not show fluorescence (Figure 6B), suggesting that in the context of the riboswitch, AUG1 is not used for the start of translation. By contrast and as already shown in Figure 4B above, cultures containing pCD33-egfp (that corresponds to a transcript harbouring AUG1 and AUG2) showed fluorescence (Figure 6B). To test which AUG is the start codon in the reporter transcript of pCD33-egfp, the putative start codons were mutated. When AUG1 was mutated to UAG (pCD33-AUG1m-egfp; Figure 6C and 6D), fluorescence was still observed (Figure 6B). By contrast, the mutation of AUG2 to GUC (Figure 6C) abolished *egfp* expression (see pCD33-AUG2m-egfp in Figure 6B). These results suggested that in the natural context, AUG2 is the translational start site.

Next, we asked whether the SD sequence GAGG upstream of AUG1, which seems to belong to the expression platform of the SAM-II riboswitch (Figure 6D and Figure 1B above), plays a role in *metA* expression. In the reporter plasmid pCD33-egfp, the SD sequence was mutated from GAGG to GATT (Figure 6C and Figure 6D). This mutation essentially abolished *egfp* expression (compare pCD33-SDm-egfp to pCD33-egfp in Figure 6B), showing that the SD sequence in the riboswitch is required for *metA* expression.

### The metA SAM-II riboswitch regulates RNA stability and translation in an opposite manner

**Figure 7.**
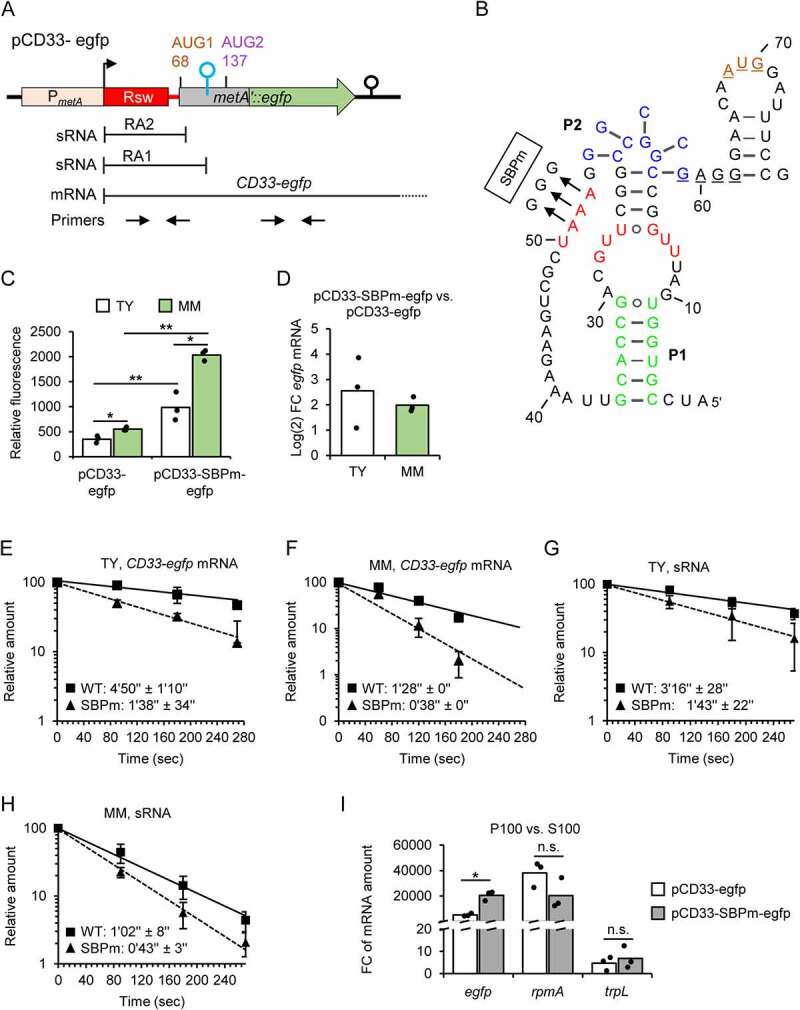
The *metA* SAM-II riboswitch regulates RNA stability and translation in an opposite manner. A) Schematic representation of the translational fusion plasmid pCD33-egfp with sRNA and *CD33-egfp* mRNA products, and primer pairs used for qRT-PCR analysis. B) Predicted secondary structure of the riboswitch in its SAM-bound form (compare to Figure. 1B). Shown is the SBPm mutation present in the recombinant sRNAs and *CD33-SBPm-egfp* mRNA transcribed from pCD33-SBPm-egfp. C) Relative fluorescence of TY and MM cultures of *S. meliloti* 2011 strains carrying the indicated plasmids. D) Comparison of the *CD33-egfp* and *CD33-SBPm-egfp* mRNA levels in TY and MM cultures of *S. meliloti* 2011 strains harbouring the respective plasmids, shown as log_2_FC. The mRNA levels were determined by qRT-PCR using *egfp*-specific primers (see panel A). E) to H) Before and after rifampicin addition to TY or MM cultures (indicated), RNA was purified from *S. meliloti* 2011 (panels E and F) or the 2011 ΔRA mutant (panels G and H) harbouring either pCD33-egfp (WT) or pCD33-SBPm-egfp (SBPm). qRT-PCR was performed to determine mRNA half-lives (using *egfp*-specific primers) or sRNA half-lives (using sRNA-detecting primers; see panel A). Half-life determination of sRNAs with the sRNA-detecting primers is possible, because the cellular sRNA levels are much higher than the mRNA levels (see Figure S7). For further details, see Figure. 1A and Figure. 3. I) Comparison of the relative levels of the *CD33-egfp* and *CD33-SBPm-egfp* mRNA in P100 and S100 fractions of *S. meliloti* 2011 strains, each containing the respective plasmid, using qRT-PCR. The ribosomal protein L27 encoding mRNA *rpmA* and the dual function sRNA rnTrpL, which harbours the small ORF *trpL* and is translated as a leaderless small mRNA, were used as controls. All graphs show means and single data points or s.d. of three independent experiments.

To address the role of the SAM-II riboswitch in regulating *metA* expression, we decided to mutate the SAM-binding pocket (SBP) of the reporter fusion mRNA transcribed from pCD33-egfp. Cultures of *S. meliloti* 2011 strains harbouring either plasmid pCD33-egfp or its derivative pCD33-SBPm-egfp that carries the SBP mutation (Figure 7A and Figure 7B) were compared in terms of fluorescence, steady state mRNA levels, mRNA stabilities and mRNA association with ribosomes.

The SBP mutation, which is expected to abolish SAM binding by the riboswitch (Figure 7B), led to increased fluorescence of the cultures in both media (Figure 7C). This was paralleled by a higher level of the mutated fusion mRNA *CD33-SBPm-egfp* when compared to the level of *CD33-egfp* mRNA, as estimated by qRT-PCR using an *egfp*-specific primer pair (Figure 7A and Figure 7D). The increase in the mRNA level caused by the SBP mutation could result from 1) increased P*_metA_* activity if the SBP-sequence corresponds to a transcription factor-binding site, 2) increased read-through transcription at the terminator shown in Figure 4D above and/or 3) increased mRNA stability, although for the latter we expected an opposite effect. We determined the mRNA half-lives and found that in both media, the SBP mutation destabilized the fusion reporter mRNA (Figure 7E and 7F). This is in line with the proposed role of the SAM-II riboswitch in stabilizing mRNA when bound to its ligand (as mentioned in the introduction), but also shows that the transcriptional effect of the SBP mutation is strong, since under both growth conditions, the levels of the mutated mRNA were increased despite its destabilization (see Figure 7D to Figure 7F).

Next, we wished to analyse the half-lives of the plasmid-born sRNAs, in order to confirm the role of the SAM-II riboswitch as RNA stability determinant. To this end, *S. meliloti* 2011 ΔRA mutant with chromosomal deletion of the SAM-binding aptamer sequence was constructed (Figure S7A to S7C). This mutant strain does not have chromosome-born transcripts that could be detected with the primer pair targeting the sRNAs RA1 and RA2, and the 5’-UTR of *metA* (Figure 7A). The SAM level in the 2011 ΔRA mutant was similar to the level in the parental strain in both TY and MM (Figure S7D). Plasmids pCD33-egfp and pCD33-SBPm-egfp were conjugated into the ΔRA strain, and total RNA samples were analysed by qRT-PCR with the two primer pairs depicted in Figure 7A to address the relative levels of the plasmid-born sRNAs and the read-through mRNA. This analysis confirmed the much higher level of the sRNAs in comparison to the read-through mRNAs (ΔCq = 7 cycles; Figure S7E). Thus, the sRNA-detecting primers depicted in Figure 7A should be useful to address the stabilities of the plasmid-born sRNAs. The results of the half-life determination using the sRNA-detecting primers confirmed that the SBP mutation leads to transcript destabilization (Figure 7G and Figure 7H). We conclude that the SBP is a stability determinant not only for the mRNAs but also for the SAM-II riboswitch containing sRNAs.

Finally, to address the role of the SAM-II riboswitch in regulating *metA* translation (see Figure 1B above), we compared the proportions of ribosome-bound *CD33-egfp* and *CD33-SBPm-egfp* mRNAs in *S. meliloti* 2011 strains harbouring the corresponding plasmids and grown in TY medium. Cultures were treated to trap the ribosomes on mRNA (see Methods), and cleared lysates were fractionated by ultracentrifugation at 100,000 g. The mRNA level in the pellet (P100; contains ribosomes and polysomes) was compared to that in the supernatant fraction (S100; corresponds to ribosome-free mRNA) by qRT-PCR using the *egfp*-directed primers. Even though the vast majority of the *CD33-egfp* mRNA was in P100, the proportion of the *CD33-SBPm-egfp* mRNA in P100 was significantly higher (Figure 7I). For the control genes *rpmA* and *trpL* [28], higher proportion of ribosome-bound mRNA in strain 2011 pCD33-SBPm-egfp was not detected, showing that this is a specific feature of the *CD33-SBPm-egfp* mRNA (Figure 7I). These results suggest that ribosome binding to *metA* mRNA is increased when the SAM-II riboswitch is in its ligand-free form.

Together, the results shown in Figure 7 suggest that under high SAM conditions, the SAM-II riboswitch downregulates *metA* translation and concomitantly stabilizes the mRNA.

## Discussion

The genes *metA* and *metZ* are involved in the biosynthesis of methionine and thus also SAM, which is essential for the bacterial metabolism and gene regulation [2]. In this work, we show that in *S. meliloti*, their expression is regulated at the transcriptional and post-transcriptional level. In *E. coli*, adjustment of the SAM level to the cellular needs strongly relies on the MetJ repressor that controls the transcription of most *met* genes (reviewed in [29]). MetJ is a homodimer, which needs SAM as a co-repressor for efficient binding to so-called *met*-boxes on the DNA [30–33]. This mechanism is the basis of the commercial assay, which we used to determine the SAM concentration in bacterial lysates (see Material and Methods). However, *S. meliloti* lacks a MetJ homolog, and the mechanism for the increased activity of the *metA* and *metZ* promoters in minimal medium (Figure 4 and Figure S6), under low methionine and SAM conditions, remains to be elucidated.

Our work suggests that at the post-transcriptional level, in *S. meliloti* the *metA* and *metZ* genes are regulated by SAM-II riboswitches with dual functions. In line with the high conservation of the SAM-II riboswitches in Alphaproteobacteria [8], the two *S. meliloti* riboswitches have very similar but not identical aptamers (Figure S3). Upon SAM-binding, the aptamers were predicted to adopt pseudoknot structures (Figure 1B, Figure S1). Thus, in the read-through mRNAs needed for *metA* and *metZ* expression, a pseudoknot could be formed in the riboswitch-containing 5′-UTRs. As mentioned in the introduction, such a ligand-induced pseudoknot was shown to stabilize mRNA in *L. pneumophila* by limiting the access of 5′-bound RNase E to its cleavage sites along the transcript [16]. This is in line with the observed *metA* and *metZ* mRNA stabilization in *S. meliloti* under high SAM conditions in TY medium (Figure 3E and Figure 3F). Additionally, the importance of the SAM-binding pocket for *metA* mRNA stabilization was shown by its mutation in a reporter fusion transcript (Figure 7E and Figure 7F). The SBP mutation also increased the ribosome association and thus probably the translation of the reporter transcript (Figure 7I). Usually, higher ribosome occupancy is expected to stabilize mRNA [34], but our results suggest the opposite for *metA*. A function of the SAM-II riboswitches in mRNA stabilization under high SAM conditions is contra intuitive but could contribute to SAM homoeostasis, as discussed below.

By contrast, the originally proposed functions of the riboswitches in transcription or translation are in line with the expected *metA* and *metZ* downregulation under high SAM conditions. Upon SAM binding, rearrangement in the expression platform of the *metZ* riboswitch most probably causes premature transcriptional termination (Figure S1). This is in agreement with a previously observed downregulation of expression of a translational *metZ’::egfp* fusion upon ectopic induction of *metK* expression and thus SAM availability [35]. For the *metA* riboswitch, the prediction suggests that upon SAM binding, the SD sequence in the expression platform of the *metA* riboswitch becomes inaccessible (Figure 1B). This was supported by our experimental data showing that the SBP mutation increased the mRNA interaction with ribosomes (Figure 7I). Thus, under high SAM conditions, both riboswitches deploy different post-transcriptional mechanisms to downregulate *metA* and *metZ* expression, acting coherently with the decrease in the P*_metA_* and P*_metZ_* promoter activities. As mentioned above, this downregulation is simultaneously counteracted by stabilization of the *metA* and *metZ* mRNAs by the riboswitches, which thus exert incoherent dual functions.

Why was the incoherent dual regulation by the SAM-II riboswitches developed in evolution? Bacterial mRNAs are generally unstable, but with half-lives of approximately 30 sec in MM (Figure 3E and Figure 3F), the *metZ* and *metA* mRNAs belong to the least stable mRNAs [36]. Such an instability under high SAM conditions, when promoter activity is downregulated (and, in the case of *metZ*, transcription of the gene is prematurely abolished), would rashly lead to mRNA depletion. This would be disadvantageous for fast adaptation to a sudden change from high to low SAM conditions. The more than threefold longer half-lives of the *metZ* and *metA* mRNAs in TY than in MM probably serve to buffer the decrease in their steady-state levels, thus ensuring SAM homoeostasis and adaptation to changing environmental conditions.

Very short half-lives in MM were also determined for the riboswitch-containing sRNAs, which were also stabilized in TY medium (Figure 3B to Figure 3D), most probably upon SAM binding (Figure 7G and Figure 7H). Due to this stabilization (and in the case of the RZ sRNA, due to increased transcriptional termination), the RZ and RA2 sRNAs accumulated and the level of the RA1 sRNA (which is most probably processed to RA2) remained similar to that in MM despite the decreased transcription from P*_metZ_* and P*_metA_* in TY medium. The RZ and RA2 accumulation raises the question of whether these sRNAs have functions in *trans* under high SAM conditions. For several 5′-UTR-derived sRNAs functions in *trans* were described [24,37–39]. It is tempting to speculate that the RZ and RA2 riboswitch sRNAs could interact with MetK, which was recently proposed to act as an RNA binding protein [35], and regulate its activity. However, we were not able to detect specific interactions between FLAG-MetK and RZ, RA1 or RA2 by coimmunoprecipitation (unpublished data).

The presence of a transcriptional terminator downstream of the translation-controlling *metA* riboswitch (between AUG1 and AUG2) was surprising. Previously, tandem riboswitches were reported [40]. However, we could not predict a second riboswitch containing the terminator or a SAM-II riboswitch-driven antiterminator. Although our data strongly suggest an increased transcription into the *metA* gene upon mutation in the SBP sequence (Figure 7D to Figure 7F), it is not clear whether this is due to increased promoter activity or decreased post-transcriptional termination. However, the data clearly show that this terminator drastically downregulates *metA* expression in both TY and MM. In both media, the relatively strong transcription from P*_metA_* combined with strong premature transcription termination leads to higher production of the RA1 (and thus also RA2) sRNA than of the read-through *metA* mRNA (Figure 2D and Figure 4B). This suggests that a major function of the terminator could be to ensure the sRNA production, reinforcing the idea that RA1 and/or RA2 may have function in *trans*, a possibility that remains to be tested in the future.

Our most surprising finding was the *in vivo* usage of AUG2 instead of AUG1 for initiation of *metA* translation. According to Figure 1B and Figure 5, the SAM-binding aptamer of the *metA* riboswitch is followed by an RBS composed of an SD sequence and AUG1, which is competent to drive translation when used outside of its natural context. However, in the context of the 5′-UTR of *metA* only the SD was required to start *metA* translation 74 nt downstream at AUG2 (Figure 6). Strikingly, outside of its natural context, AUG2 was not capable of mediating translation initiation (Figure 5) but was crucial for translation of the read-through *metA* mRNA harbouring its natural 5′-UTR (Figure 6). A possible interpretation of these results is that the SD of the riboswitch is used to recruit the 30S ribosomal subunit, which, however, cannot interact with AUG1, probably because of the two- and/or three-dimensional structure of the *metA* 5′-UTR (which was not analysed experimentally). Instead, the recruited 30S subunit seems to form a translation initiation complex at AUG2. Most probably, the role of the SD in the riboswitch is to increase the local concentration of the 30S subunit and thus the probability to start translation at AUG2.

The proposed new mechanism for translation initiation is different from the known standby mechanisms, which are based on 30S subunit binding upstream of a translation initiation region (TIR) without RNA sequence specificity [41]. Instead, the S1 protein is usually involved in the ribosome loading on a standby site [42–44]. Recently, the S1 protein was shown to bind to a pseudoknot in the standby site of *tisB* mRNA, the distant TIR of which is sequestered in a secondary structure [45]. The situation in the 5′-UTR of *metA* mRNA is different since our results suggest that the pseudoknot formation in the SAM-bound state downregulates translation by reducing the accessibility of the SD sequence in the expression platform of the riboswitch (Figure 1B, Figure 7I). Our discovery that translation initiation can depend on a distant SD sequence complicates the task of identifying the translation start of a gene. It is not known how widespread this mechanism is among SAM-II regulated genes.

**Figure 8.**
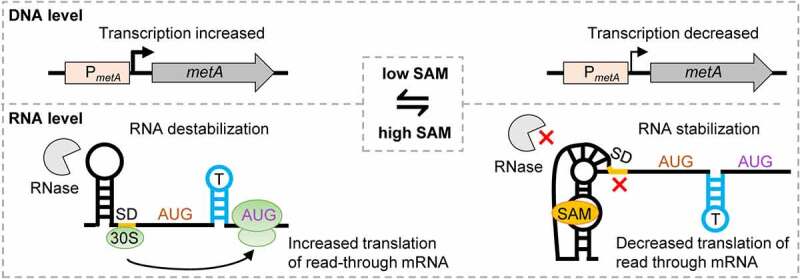
Model of *metA* regulation at the transcriptional and post-transcriptional level. On the left: Under low SAM conditions, transcription from P*_metA_* is increased, the riboswitch is in the SAM-free state, the SD controlled by the riboswitch is accessible and activates translation in the read-through mRNA from the starting codon AUG2. Under these conditions, *metA* mRNA is destabilized, probably due to RNase access to specific cleavage site(s) in the mRNA. On the right: Under high SAM conditions, transcription from P*_metA_* is decreased, the riboswitch is in the SAM-bound state, the SD controlled by the riboswitch is not accessible and *metA* translation is repressed. Under these conditions, *metA* mRNA is stabilized, probably because the riboswitch pseudoknot structure blocks the RNase access [16].

Our results strongly suggest that in *S. meliloti*, the SAM-II riboswitches of *metA* and *metZ* mediate incoherent dual regulation, which aims to decrease gene expression under high SAM conditions, without depleting the mRNAs to unfavourable levels. A model for the *metA* regulation summarizing our data is presented in Figure 8. The model also shows the suggested novel mechanism for translation initiation, which is based on a riboswitch-controlled Shine–Dalgarno sequence acting as a standby site.

## Material and methods

### Strains cultivation and conjugation

*Sinorhizobium (Ensifer) meliloti* 2011 [46] was cultivated in TY [47] or minimal GMS [48] medium (MM) with appropriate antibiotics (streptomycin 250 μg/ml; gentamicin 10 µg/ml; neomycin 120 µg/ml). Liquid cultures were grown under semi-aerobic conditions (30 ml in 50 ml Erlenmeyer flask at 30°C, 140 rpm) until OD_600nm_ 0.5 unless stated otherwise.

To obtain strain 2011 *FLAG-metK*, the integration plasmid pKACIT-FLAG-metK was used. A manuscript dealing with the construction and testing of this plasmid is under preparation (J. Kretz and M. McIntosh). Briefly, this plasmid harbours the *lacI* repressor gene under the control of the *eil* system (Jungle Express) which uses crystal violet as the inducer [49]. Furthermore, it contains an IPTG-inducible promoter and an RBS, the start codon of which is followed by 3× FLAG and *metK* codons. Plasmid integration places the essential gene *metK* (encoding the SAM synthetase) under the control of the IPTG-inducible promoter and tags the MetK protein at the N-terminus with 3× FLAG. During conjugation of this plasmid to *S. meliloti*, 1 mM IPTG was added to the TY medium to ensure *FLAG-metK* expression in conjugants with integrated plasmid and thus their viability. Pre-cultures of the strain 2011 *FLAG-metK* were grown in TY with 1 mM IPTG overnight. The next day, the cultures were diluted in TY to an OD_600 nm_ of 0.2 and grown to an OD_600 nm_ of 0.5 with 0.2 µM crystal violet to induce *lacI* expression and without IPTG (to deplete the SAM synthetase) or with different IPTG concentrations (0.1, 0.5 and 1 mM) to generate a gradient in the levels of FLAG-MetK in the cells.

*Escherichia coli* was grown in LB broth. *E. coli* DH5α (Thermo Fisher Scientific) was used for standard cloning methods [50]. Plasmids were transferred from *E. coli* S17-1 to *S. meliloti* by diparental conjugation [51].

### Plasmid construction and chromosomal modification

The plasmids used in this work are listed in Table S1 and the oligonucleotides (synthesized by Microsynth, Balgach, Switzerland) in Table S2. Cloning procedures were conducted as described [50]. Enzymes and the CloneJet PCR Cloning Kit were purchased from Thermo Fisher Scientific. After cloning in plasmid pJET1.2/blunt, inserts were subcloned into the conjugative plasmid pSRKGm [52] or its derivative pRS1. The final constructs were routinely sequenced with plasmid-specific primers (sequencing service by Microsynth Seqlab, Göttingen, Germany).

To obtain pRS1, the *lac* module of pSRKGm was cut out using the restriction endonucleases NheI and EcoRI and was replaced by a synthetic multiple cloning site. In the EcoRI restriction site, a sequence corresponding to the transcriptional terminator of the *B. japonicum* USDA 110 *rrn* genes (T_*rrn*_) was cloned [53], resulting in plasmid pRS1-T.

To generate pP_metA_-egfp, first *egfp* was amplified using pLK64 [54] as a template and a forward primer harbouring the SD sequence GAGGAG nine nucleotides upstream of the *egfp* start codon. The insert was cloned into pRS1-T using the XbaI and BamHI restriction sites, resulting in pRS1-SD-egfp. Afterwards, the P_*metA*_ region from −209 to +5 (+1 is the TSS [27];) was amplified and cloned between the NheI and XbaI restriction sites of pRS1-SD-egfp. The plasmid pP_metZ_-egfp, which contains the *metZ* region from −206 to +5, was constructed similarly.

To construct pTerm-egfp, synthetic oligonucleotides Term-se and Term-as, which correspond to the terminator sequence located between AUG1 and AUG2 of *metA* (Figure 1A), were annealed and cloned into the XbaI site of pP_metA_-egfp. Similarly, pTerm-M1-egfp was constructed using the oligonucleotides Term-gg106,107cc-se and Term-gg106,107cc-as. To construct pTerm-M1+ M2-egfp, site directed mutagenesis of the pTerm-M1-egfp was performed by inverse PCR with Phusion polymerase, followed by DpnI treatment (Thermo Fisher Scientific).

For the construction of translational fusion plasmids, the *egfp* gene was amplified from the third codon to the stop codon and cloned between the BamHI and PstI restriction sites of pRS1-T. In the resulting plasmid pRS-′egfp, the reporter gene lacks a promoter and an RBS. To construct the plasmids pCD3-egfp, pCD16-egfp and pCD33-egfp, *metA* regions were amplified starting from position −209 and encompassing the first 3, 16 and 33 codons (AUG1 was considered as the first codon), respectively. Each amplicon was first cloned in pJET1.2/blunt and then subcloned between the restriction sites NheI and BamHI of pRS-′egfp, resulting in different sizes of truncated translational metA’::egfp fusions.

Plasmids pCD33-AUG1m-egfp, pCD33-AUG2m-egfp, pCD33-SDm-egfp and pCD33-SBPm-egfp were generated by site directed mutagenesis of plasmid pJET-CD33 and subcloning of the mutated inserts in pRS-′egfp.

Plasmid pAUG1-egfp was constructed as follows. First, pJET-CD33 was used in an inverse PCR to delete the *metA* region +13 to +58. The remaining *metA* sequence in the resulting pJET-metA-a::b was used as a template to amplify the fused *metA* regions a and b (see Figure 5). The resulting amplicon was used in an overlapping PCR to fuse it in frame to the third *egfp* codon. The final reporter fusion was cloned between the NheI and PstI sites of plasmid pRS1-T. Similarly, plasmid pAUG2-egfp was constructed. In this case, first the *metA* region from +13 to +124 was deleted by inverse PCR of pJET-CD33, resulting in pJET-metA-a::c. Then, the fused regions a and c (see Figure 5) were amplified and fused in frame with *egfp*.

To generate the integration plasmid pKACIT-FLAG-metK, approximately 400 bp of the *metK* gene were amplified starting with the second codon. Using the restriction sites XbaI and KpnI of pKACIT-FLAG, the amplicon was inserted in frame downstream of a 3× FLAG-coding sequence. Integration of the plasmid into the *metK* locus results in a recombinant *metK* gene, which is under the control of an IPTG inducible promoter and encodes an N-terminally tagged FLAG-MetK protein.

Plasmid pK18mobsacB-ΔRA was used to delete the aptamer of the *metA* riboswitch as described [55]. Chromosomal regions flanking the sequence which was deleted (from position +3 to position +59 of the transcript shown in Figure 1B) were amplified using the primers given in Table S2 and cloned in pK18mobsacB [56] between the EcoRI and Xba restriction sites. During deletion, the TY medium was supplemented with 20 µg/ml L-methionine. Successful deletion was confirmed by PCR analysis of cell lysates using primers located upstream and downstream of the deleted region and Northern blot analysis in comparison with the parental strain. Since the *metA* promoter and coding sequence were preserved, the deletion strain was able to grow in the medium without methionine.

### Estimation of SAM levels

The Bridge-IT S-Adenosyl Methionine (SAM) Fluorescence Assay Kit (Mediomics) was used to determine SAM levels in *S. meliloti*. Cells of 1 ml of TY or MM culture were harvested and washed twice with cold water. For lysis and SAM estimation, the cells were treated according to the manufacturer’s instructions and as described previously [57]. The bacterial pellet was resuspended in 40 µl SAM extraction buffer and treated with the assay solution. After 1.5 h incubation at room temperature, sample fluorescence (F) and fluorescence of the assay solution (F0) were measured (wavelength settings: absorption 485 nm; emission 665 nm). Relative sample fluorescence was calculated $\frac{F-F0}{F0}$ and set in relation to the calibration curve, which was obtained using a 1:2 serial dilution of a sample with known SAM concentration.

### Determination of cell length and cell count

To estimate the cell length, an image analysis, microscope Leica DMI6000B (Leica), the phase-contrast objective HCX PL APO 100x/1.4 PH3 (Leica), and the software tool BacStalk was used [58], and 350 cells per condition (TY or MM) were considered. The cell count per ml TY and MM was estimated using an improved Neubauer counting chamber (Marienfeld Superior) with a chamber depth of 0.1 mm. Cells were counted in four squares with an area of 0.025 mm^2^ in order of a ‘Z.’ Cells on the bottom and right boundaries were not counted.

### RNA isolation and analysis

If not stated otherwise, RNA was isolated using TRIzol® reagent (Thermo Scientific, Waltham, USA) as previously described [24], with the following modifications. Cell pellet from a 15 ml *S. meliloti* culture (OD_600 nm_ = 0.5) was resuspended in 1 ml TRIzol. Then, 1 µl of a spike-in control transcript (1 ng/µl) was added, and RNA was isolated according to the manufacturer’s instructions. After RNA precipitation and dissolving in cold ultrapure water, additional extractions with hot acidic phenol and chloroform-isoamyl alcohol (24:1) were conducted. Then, the RNA was ethanol precipitated, washed with 75% ethanol, dried and dissolved in ultrapure water. Concentration and purity of RNA were estimated by absorbance measurement at 260 nm and 280 nm. RNA integrity was analysed in a 10% polyacrylamide-urea gel stained with ethidium bromide. For the comparison of the steady-state levels of sRNAs and mRNAs, total RNA was purified by the hot phenol method [25]. For estimating the *egfp* mRNA half-lives (see below) in strains containing PmetA-egfp, pCD33-egfp, or pCD33-SBPm-egfp, cultures were treated with RNAprotect Bacteria Reagent (Qiagen) before the cells were harvested, and RNA was isolated with the RNeasy Mini Kit (Qiagen) according to the manufacture’s instructions.

Northern blot hybridization was performed to analyse sRNA levels as described [59]. Total RNA was separated in a 10% polyacrylamide-urea gel in a TBE buffer at 300 V for 4 h. Using a Semi-Dry Blotter, RNA was transferred to a positively charged nylon membrane for 2 h at 150 mA. The membrane was incubated for 2 h at 56°C in pre-hybridization buffer (6× SSC, 0.5% SDS, 10 µg/ml Salmon Sperm DNA, and 2.5× Denhardt’s solution). For hybridization with radioactively labelled oligonucleotides (Table S2), the membrane was incubated for at least 6 h at 56°C in hybridization-buffer (6× SSC, 0.5% SDS and 10 µg/ml Salmon Sperm DNA, to which 5 µl of the labelled oligonucleotide probe was added). The membrane was washed at room temperature three times for 1 min in 0.01% SDS, 5× SSC. A Bio-Rad molecular imager and the Quantity One (Bio-Rad) software were used to detect and quantify the signals. The 5S rRNA was used as loading control.

For analysing steady-state mRNA levels by quantitative real-time reverse transcriptase-polymerase chain reaction (qRT-PCR), first residual DNA was removed by treatment of 10 µg RNA with 1 µl TURBO-DNase for 30 min. For each RNA sample, the success of the treatment was tested by PCR with *rpoB*-specific primers. The qRT-PCR was performed by using Brilliant III Ultra Fast SBR® Green QRT-PCR Master Mix (Agilent), a spectrofluorometric thermal cycler (Bio-Rad), and, for quantification, the program BioRad CFX Manager 3.0, as described previously [60] with the following change: an external spike-in control RNA, *crtA* (see below), was used as a reference. The cycle, at which the amplification maximum of the curvature was reached, was set as quantification cycle (Cq). Fold changes of mRNA amounts were calculated using the Pfaffl-formula and the respective Cq-values of genes of interest and the spike-in control. The primer pair efficiencies were determined by PCR using serial two-fold dilutions of RNA (Table S2). All qRT-PCR analyses were done in three independent experiments with technical duplicates.

To synthesize *crtA* spike-in RNA by *in vitro* transcription using the MEGAscript™ T7 Transcription Kit (Thermo Fisher Scientific), a 718 bp region of the gene *crtA* of *Rhodobacter sphaeroides* was amplified, introducing a T7 promoter by the forward primer. In an *in vitro* transcription reaction of 20 µl, 500 ng of the amplicon were used, which was purified by using the MinElute PCR Purification Kit (QIAGEN). The GC-content of *R. sphaeroides* is similar to that of *S. meliloti*, but *S. meliloti* has no *crtA* homolog.

To estimate the RNA half-lives, transcription was terminated by rifampicin (Rif) addition (final concentration of 600 µg/ml) to 300 ml of *S. meliloti* cultures grown in 500 ml Erlenmeyer flasks at 30°C and 140 rpm to an OD_600 nm_ of 0.5. Culture samples (15 ml) were withdrawn before (t = 0) and at the indicated time points after Rif addition. After RNA isolation, qRT-PCR and Northern blot analysis were performed as described above. To calculate the half-lives, linear-log graphs were used. The RNA amount at the time point 0 was set to 100%.

To estimate the length of the riboswitch sRNAs, the membrane was hybridized sequentially with probes detecting RZ, RA1 and RA2 and afterwards with tRNA^Thr^ (86 nt), 6S RNA (156 nt) and 5S rRNA (120 nt). The migration distance was measured, and a linear-log graph was used to calculate the length of the sRNAs.

Models of RNA structures were predicted with RNAfold [61].

### Analysis of the relative amounts of ribosome-bound mRNA

Cultures of *S. meliloti* 2011 harbouring either pCD33-egfp or pCD33-SBPm-egfp were grown in TY to the OD_600 nm_ of 0.5 and rapidly chilled in an ice-water bath. The cells were harvested by centrifugation (10 min at 3,500 g and 4°C) and frozen in liquid nitrogen. The cell pellets were resuspended in a cold buffer containing 1 M NH_4_Cl, 150 mM MgCl_2_, 20 mM Tris-HCl, pH 8.0, 5 mM CaCl_2_, 0.4% Triton X-100, 150 U DNase I, 1000 U RNase inhibitor and lysed by sonication. After centrifugation of the lysate at 10,000 g and 4°C for 12 min, the supernatant (cleared lysate) was subjected to ultracentrifugation at 100,000 g and 4°C for 1 h. RNA was isolated from the supernatant (S100) and the pellet (P100) fractions using the hot phenol method. The precipitated RNA of each fraction was dissolved in the same volume of ultrapure water, and *crtA* spike-in transcript was added to each RNA sample. The P100 fraction was diluted and 40 ng, 20 ng and 10 ng RNA was used in qRT-PCR reactions with *egfp*-specific primers to validate the dynamic range of detection of the reporter mRNA. Identical dilutions and volumes of S100 RNA samples were also subjected to qRT-PCR and the linear relationship between *egfp* template and Cq values in the diluted samples was validated. Finally, 40 ng RNA of the P100 fraction and accordingly diluted S100 RNA were used to compare the relative levels of *CD33-egfp, CD33-SBPm-egfp, rpmA* and *trpL* mRNAs in the S100 and P100 fractions by qRT-PCR using *crtA* as a control RNA.

### Oligonucleotides labelling

For radioactive labelling, 10 pmol of an oligonucleotide (Table S2) was 5′ phosphorylated by [^32^P]-adenosine-5′-triphosphate (3000 Ci/mmol, 10 mCi/ml) using 5 U of T4-polynucleotide-kinase and the buffer A provided by the manufacturer (NEB). The 10 µl reaction mixture was incubated for 1 h at 37°C. Reaction was stopped by adding 40 µl STE-buffer (10 mM Tris-HCl pH 8, 0.1 M NaCl, 2 M EDTA). Unbound nucleotides were removed using a G25-column (GE Healthcare).

### Western blot analysis

Production of the fusion FLAG-MetK protein was tested by Western blot analysis. Cells were harvested by centrifugation at 4°C and resuspended in a sample buffer [62]. Before loading on a gel, samples were heated to 95°C for 10 min. Samples corresponding to an OD_600 nm_ of 1 were separated in 10% polyacrylamide gels by SDS-PAGE. Proteins were transferred to a polyvinylidene difluoride (PVDF) membrane using a Semi-Dry Blotter. For protein detection, monoclonal, horseradish peroxidase-conjugated antibody raised against the FLAG tag (Sigma Aldrich) and Western Lightning Ultra, Chemiluminescent Substrate (PerkinElmer) were used. To visualize the signals, a Fusion-SL chemiluminescent imager (Peqlab) was used.

### Fluorescence measurement

*S. meliloti* conjugants containing *egfp* reporter plasmids were cultivated in TY or MM to an OD_600 nm_ of 0.5, and 150 µl of the cultures were transferred to a 96-well microtiter plate. Fluorescence was measured using a Tecan Infinite M200 reader. Values were normalized to the measured OD_600 nm_ and the autofluorescence of the empty vector control culture. All measurements were done in three independent replicates with technical triplicates.

### Statistical analysis

If not stated otherwise, all data are shown in means and single data points of three independent experiments. The Welch’s *t-*test was used for statistical analysis. Differences were not considered significant if *P* value was >0.05. One asterisk symbolizes *P* values ≤ 0.05 and two asterisks correspond to *P* values ≤ 0.005.

## Supplementary Material

Supplemental Material

## Data Availability

The authors confirm that the data supporting the findings of this study are available within the article and its supplementary materials.
