# Supplementary material for: Incoherent dual regulation by a SAM-II riboswitch controlling translation at a distance
Source: RNA Biol. 2022 Aug 11;19(1):980–95. doi: 10.1080/15476286.2022.2110380 (PMC9373788; doi:10.1080/15476286.2022.2110380)
Supplement: Supplemental Material [file KRNB_A_2110380_SM3921.zip › Table_S1_Plasmids_used_in_this_work.docx]

| **Table S1 Plasmids used in this work** | |  |
| --- | --- | --- |
|  |  |  |
| **Plasmid*** | **Description** | **Reference** |
| pJET1.2/blunt | Cloning vector, Amp^r^ | Thermo Fischer Scientific |
| pLK46 | Contains *egfp* with GC rich codons; Tc^r^ | McIntosh et al., 2008 |
| pJet-CD33 | pJET1.2/blunt derivative; contains the *metA* region starting from -209 (+1 is the TSS) and encompassing the first 33 codons (ATG1 is considered as the first codon). | This work |
| pJET-metA-a::b | pJet-CD33 derivative, the *metA* sequence from +13 to +58 (+1 is the TSS) was deleted by inverse PCR. | This work |
| pJET-metA-a::c | pJet-CD33 derivative, the *metA* sequence from +13 to +124 (+1 is the TSS) was deleted by inverse PCR. | This work |
| pKACIT-FLAG | Integration vector with IPTG incucible promoter and 3×FLAG-tag codons for N-terminal fusion; Nm^r^ | Kretz J and McIntosh M, manuscript in preparation. |
| pKACIT-FLAG-metK | pKACIT derivative; contains 400 nt of the *metK* sequence between the XbaI and KpnI restriction sites. The *metK* sequence, starting from the second codon, was cloned in frame with the 3×FLAG tag codons. | This work |
| pK18mobsacB | Suicide plasmid with *sacB* lethal in the presence of sucrose in the medium; Km^r^ | Schäfer et al., 1994 |
| pSRKGm | Broad host range expression vector with IPTG inducible P*_lac_*; Gm^r^ | Khan et al., 2008 |
| pRS1 | pSRKGm derivative; the *lac* module *was* replaced by a synthetic MCS harbouring restriction sites for NheI, HindIII, XbaI, SpeI, BamHI, PstI, and EcoRI; Gm^r^ | This work |
| pRS1-T | pRS1 derivative; the transcriptional terminator of the *B. japonicum* USDA 110 *rrn* genes was cloned into the EcoRI restriction site. | This work |
| pRS1-SD-egfp | pRS1-T derivative; a Shine-Dalgarno sequence and *egfp* were cloned between the XbaI and BamHI restriction sites. | This work |
| pP_metZ_-egfp | pRS-SD-egfp derivative; promotor fusion plasmid; contains the *metZ* region from -206 to +5 (+1 is the TSS) between the NheI and XbaI restriction sites. | This work |
| pP_metA_-egfp | pRS-SD-egfp derivative; promotor fusion plasmid; contains the *metA* region from -209 to +5 (+1 is the TSS) between the NheI and XbaI restriction sites. | This work |
| pTerm-egfp | pP_metA_-egfp derivative; RA1 terminator cloned into the XbaI restriction site. | This work |
| pTerm-M1-egfp | pP_metA_-egfp derivative; destabilised RA1 terminator with GG106,107CC mutation cloned into the XbaI restriction site. | This work |
| pTerm-M1+M2-egfp | pTerm-M1-egfp derivative; contains compensatory CC83,84GG mutation restoring the RA1 terminator. | This work |
| pRS-'egfp | pRS1-T derivative; harbors truncated *egfp* starting from the third codon, which was cloned using BamHI and PstI ; lacks a promotor and an RBS. | This work |
| pCD3-egfp | pRS-'egfp derivative; translational fusion plasmid containing the *metA* region starting from -209 (+1 is the TSS) and encompassing first 3 codons (ATG1 is considered as the first codon) fused to *egfp;* NheI and BamHI were used for cloning. | This work |
| pCD16-egfp | pRS-'egfp derivative; translational fusion plasmid containing the *metA* region starting from -209 (+1 is the TSS) and encompassing the first 16 codons (ATG1 is considered as the first codon) fused to *egfp*; NheI and BamHI were used for cloning. | This work |
| pCD33-egfp | pRS-'egfp derivative; translational fusion plasmid containing the metA region starting from -209 (+1 is the TSS) and encompassing the first 33 codons (ATG1 is considered as the first codon) fused to *egfp*; NheI and BamHI were used for cloning. | This work |
| pCD33-M1-egfp | pCD33-egfp derivative; harbors a GG106,107 mutation destabilising the RA1 terminator located between ATG1 and ATG2. | This work |
| pCD33-AUG1m-egfp | pCD33-egfp derivative; harbors a AT68,69TA mutation in *metA;* ATG1 is mutated to a stop codon. | This work |
| pCD33-AUG2m-egfp | pCD33-egfp derivative; harbors a A137C, G139C mutation in *metA;* ATG2 is mutated to a leucin codon. | This work |
| pCD33-SDm-egfp | pCD33-egfp derivative; harbors a GG61,62TT mutation in the Shine-Dalgarno sequence of the *metA* riboswitch. | This work |
| pCD33-SBPm-egfp | pCD33-egfp derivative; harbors a AAA51,52,53TTT mutation in the SAM-binding pocket of the *metA* riboswitch. | This work |
| pAUG1-egfp | pRS1-T derivative; translational fusion plasmid designed to test whether ATG1 can drive translation. The cloned sequence lacks the *metA*aptamer. It contains P*_metA_*, the SD of the expression platform of the riboswitch, ATG1 and the following codon fused to the third *egfp* codon. The construct was obtained by overlapping PCR as described, and cloned using NheI and PstI. | This work |
| pAUG2-egfp | pRS1-T derivative; translational fusion plasmid designed to test whether ATG2 can drive translation. It contains P*_metA_*, a putative SD sequence, ATG2 and the following codon of *metA* fused to third *egfp* codon. The construct was obtained by overlapping PCR as described and cloned using NheI and PstI. | This work |
| pSRKGm-T | pSRKGm derivative; contains the *rrn* terminator sequence from *B. japonicum* USDA 110, which was cloned in the HindIII restriction site. | This work |
| pSRKGm-egfp-T | pSRKGm-T derivative; contains a Shine-Dalgarno sequence and *egfp* between the BamHI and PstI restriction sites; Gm^r^ | This work |
| pK18mobsacB-ΔRA | pK18mobsacB derivative for deletion of the aptamer of the *metA* riboswitch | This work |
| * The inserts of the most plasmids created in this work were pre-cloned in pJET1.2/blunt. Here, only pJET1.2/blunt-derivatives relevant for the pATG1-egfp and pATG2-egfp cloning are listed. | | |
