# Supplementary material for: Incoherent dual regulation by a SAM-II riboswitch controlling translation at a distance
Source: RNA Biol. 2022 Aug 11;19(1):980–95. doi: 10.1080/15476286.2022.2110380 (PMC9373788; doi:10.1080/15476286.2022.2110380)
Supplement: Supplemental Material [file KRNB_A_2110380_SM3921.zip › Table_S2_Oligonucleotides_used_in_this_work.docx]

| **Oligonucleotide designation** | **Sequence** | **Comment** | **Reference** | **Primer pair efficiency*** |
| --- | --- | --- | --- | --- |
| NB RswA-Probe 1 | ATCGCGACCGGTCCTCGGAAATC | Northern blot analysis of *S. meliloti metA* riboswitch sRNA | this work |  |
| NB RswA-Probe 2 | GCCACGTTAAAGAAGTCGCTAAAGGG | Northern blot analysis of *S. meliloti*  *metA* riboswitch sRNA | this work |  |
| NB RswZ | GCCACGTTAAACAAGTAGCTAAAAAGG | Northern blot analysis of *S. meliloti*  *metZ* riboswitch | this work |  |
| NB 5S rRNA | GTTCGGAATGGGAACGGGTGCAG | Northern blot analysis of *S. meliloti* 5S rRNA | Baumgardt et al., 2016 |  |
| Spike T7-RsCrtA-Fw | TAATACGACTCACTATAGGGCCGAATCGCCGATCTAC | For in vitro transcription template; forward primer for amplification of 600 bp of *R. sphaeroides crtA;* contains T7 promoter sequence | this work |  |
| Spike-Rs CrtA-Rv | GGGCTTGCCCTTGAAGTT | For in vitro transcription template; reverse primer for amplification of 600 bp of *R. sphaeroides crtA* | this work |  |
| qRT metA-fwd | CAACAAGATCAAGACCGAGA | *S. meliloti metA* qRT-PCR analysis, forward primer | this work | 1.964 |
| qRT metA rev | CAACTCCTTCCAGTAGGTGA | *S. meliloti metA* qRT-PCR analysis, reverse primer | this work |  |
| qRT metZ fwd | ACACCAAGGTCTTCTTCCTC | *S. meliloti metZ* qRT-PCR analysis, forward primer | this work | 1.992 |
| qRT metZ rev | CGTCTACCCACTGCTTGTC | *S. meliloti metZ* qRT-PCR analysis, reverse primer | this work |  |
| RsCrtA-qPCR-Fw | CCAGCAGGTTACCTTCTCGA | *R. sphaeroides crtA* qRT-PCR analysis, forward primer | this work | 2.009 |
| RsCrtA-qPCR-Rv | TTGCCCTCCCACTTACCAAT | *R. sphaeroides crtA* qRT-PCR analysis, reverse primer | this work |  |
| rpoB-qPCR-Fwd | ATCCTCGACACCTTCTACAC | *S. meliloti rpoB* qRT-PCR analysis, forward primer | Baumgardt et al., 2016 | 1,987 |
| rpoB-qPCR-Rev | GATAGTTGCCGTAGAGATCG | *S. meliloti rpoB* qRT-PCR analysis, reverse primer | Baumgardt et al., 2016 |  |
| qRT-metK-fwd | GATCATCGTCGACACCTATG | *S. meliloti metK* qRT-PCR analysis, forward primer | this work | 2.040 |
| qRT-metK-rev | CCTGATCTTCCGAGACTTTT | *S. meliloti metK* qRT-PCR analysis, reverse primer | this work |  |
| trpE-FW1-qPCR-19 | TCCATACCCTCGCGAACTAC | *S. meliloti trpE(G)* qRT-PCR analysis, forward primer | Melior et al., 2019 | 1.994 |
| trpE-RE1-qPCR-19 | CCTTCTTGATCGTCGCCTTG | *S. meliloti trpE(G)* qRT-PCR analysis, reverse primer | Melior et al., 2019 |  |
| qRT-RA-fwd | ATCCGTGGTGATTTGGCCG | *S. meliloti metA* riboswitch containing sRNA qRT-PCR analysis, forward primer | this work | 2.082 |
| qRT-RA-rev | ATTGTTCCCTCGGCCCTTTA | *S. meliloti metA* riboswitch containing sRNA qRT-PCR analysis, reverse primer | this work |  |
| qRT-RZ-fwd | ATCCCGTGGTGATTTGGC | *S. meliloti metZ* riboswitch containing sRNA qRT-PCR analysis, forward primer | this work | 1.931 |
| qRT-RZ-rev | TGACACCCGGCCTTTTTAGC | *S. meliloti metZ* riboswitch containing sRNA qRT-PCR analysis, reverse primer | this work |  |
| qRT-eGFP-Sme-fwd | GGACGACGGCAACTACAAGA | *egfp* (GC rich codons) qRT-PCR analysis, forward primer | this work | 2.038 |
| qRT-eGFP-Sme-rev | TTGTACTCCAGCTTGTGCCC | *egfp* (GC rich codons) qRT-PCR analysis, reverse primer | this work |  |
| RcsR1-RT-PCR-f | ATGGCAAACACGCAGAACAT | *S. meliloti* rnTrpL qRT-PCR analysis, forward primer | Melior et al., 2019 | 1.995 |
| RcsR1-RT-PCR-r | AAAGCCGCCTCGAAATCTCC | *S. meliloti* rnTrpL qRT-PCR analysis, reverse primer | Melior et al., 2019 |  |
| SMc03772_f | ATGGCACACAAGAAAGCTG | *S. meliloti rpmA* qRT-PCR analysis, forward primer | Melior et al., 2021 | 1.932 |
| SMc03772_r | GTAAGCGCAAAAATCGTATG | *S. meliloti rpmA* qRT-PCR analysis, reverse primer | Melior et al., 2021 |  |
| MSC-pSRK-se | AATTCCTGCAGGGGATCCACTAGTTCTAGAAAGCTTCTG | pRS1 construction. Sense oligonucleotide used for annealing with the anitsense oligonucleotide MSC-pSRK-as. After annealing, the dsDNA having NheI and EcoRI overhangs was used to replace the *lac*-module of pSRK-Gm by a synthetic multiple cloning site harbouring restriction sites for NheI, HindIII, XbaI, SpeI, BamHI, PstI, and EcoRI. | this work |  |
| MSC-pSRK-as | CTAGCAGAAGCTT TCTAGAACTAGTGGATCCCCTGCAG G | pRS1 construction. Antisense oligonucleotide used for annealing with the sense oligonucleotide MSC-pSRK-se | this work |  |
| Bj-Trrn-Fw-2019 | AGGCCTGCCA AGGACAAG | pRS1-T construction. Forward primer for amplification of the *rrn* terminator sequence from *B. japonicum* USDA 110 for cloning with EcoRI into pRS-1 | this work |  |
| Bj-Trrn-Rv-2019 | CGGAATTCAATTCTTGAAGACCACGGCA | pRS1-T construction. Reverse primer for amplification of *rrn* terminator sequence from *B. japonicum* USDA 110 for cloning with EcoRI into pRS-1 | this work |  |
| XbaI-sSD-egf-fw | AATCTAGATTAAAGAGGAGAAATTAACTATGGTGAGC | pRS1-SD-egfp construction. Forward primer for *egfp* amplification, with XbaI restriciton site and Shine-Dalgarno sequence | this work |  |
| BamHI-egfp-rev | AAGGATCCTTACTTGTACAGCTCGTCCATG | pRS1-SD-egfp construction. Reverse primer for *egfp*amplification, with BamHI restriciton site | this work |  |
| NheI-P_metZ_-fwd | ATTGCTAGCAAGGCGAAGATCGAGGCTG | pP_metZ_-egfp construction. Forward primer for amplification of the *metZ* region from -206 (+1 is the TSS ) to +5; contains a NheI restriction site | this work |  |
| XbaI-P_metZ_-rev | TCTAGACGGGATAAAGCTCCAATACTGCCTG | pP_metZ_-egfp construction. Reverse primer for amplification of *metZ* region from -206 (+1 is the TSS ) to +5; contains a XbaI restriction site | this work |  |
| NheI-P_metA_-fwd | AATTGCTAGCGTTTCGGGGATAAGGCAG | pP_metA_-egfp construction. Forward primer for amplification of *metA* region from -209 (+1 is the TSS ) to +5; contains a NheI restriction site | this work |  |
| XbaI-P_metA_-rev | AATCTAGACGGATCATTTGATAGCGCGAG | pP_metA_-egfp construction. Reverse primer for amplification of *metA* region from -206 (+1 is the TSS ) to +5; contains a XbaI restriction site | this work |  |
| Term-se | CTAGACGAGGACCGGTCGCGATCGTTTCGCCGCCGGTTTTTT | pTerm-egfp construction. Sense oligonucleotide used for annealing with the antisense oligonucleotide Term-as. After annealing, the dsDNA having XbaI overhangs was cloned into the XbaI site of pPmetA-egfp. | this work |  |
| Term-as | CTAGAAAAAACCGGCGGCGAAACGATCGCGACCGGTCCTCGT | pTerm-egfp construction. Antisense oligonucleotide used for annealing with the sense oligonucleotide Term-se | this work |  |
| Term-GG106,107CC-se | CTAGACGAGGACCGGTCGCGATCGTTTCGCCGCCCCTTTTTT | pTerm-M1-egfp construction. Sense oligonucleotide used for annealing with the antisense oligonucleotide RA-term-as. After annealing, the dsDNA having XbaI overhangs was cloned into the XbaI site of pPmetA-egfp. | this work |  |
| Term-GG106,107CC-as | CTAGAGGAAACCGGCGGCGAAACGATCGCGACCGGTCCTCGT | pTerm-M1-egfp construction. Antisense oligonucleotide used for annealing with the sense oligonucleotide RA-term-se | this work |  |
| Term-CC83,84GG-fwd | ACGAGGAGGGGTCGCGATCGTTTCG | pTerm-M1+M2-egfp construction. Forward primer for site directed mutagenesis CC83,84GG | this work |  |
| Term-CC83,84GG-rev | CGCGACCCCTCCTCGTCTAGACGGATC | pTerm-M1+M2-egfp construction. Reverse primer for site directed mutagenesis CC83,84GG | this work |  |
| TSS-ATG1-fwd | GGTGATGAGGGAACAATGGAT | pAUG1-egfp construction. Forward primer for inverse PCR of pJET-CD33 to obtain *metA* Δ13-58 deletion (+1 is the TSS), resulting in pJET-metA-a::b | this work |  |
| TSS-ATG1-rev | TCCCTCATCACCACGGATCATT | pAUG1-egfp construction. Reverse primer for inverse PCR of pJET-CD33 to obtain *metA* Δ13-58 deletion (+1 is the TSS), resulting in pJET-metA-a::b | this work |  |
| ATG1-egfp-fwd | ATGGATGTGAGCAAGGGCGAGGA | pAUG1-egfp construction. Forward primer for *egfp* amplfication to clone a *metA'::egfp* fusion; contains the second codon of *metA* (ATG1 was considered as the first codon) fused to the third codon of *egfp.* Used with the reverse primer PstI_egfp_rev. The amplicon was then used in an overlapping PCR with the forward primer NheI-PmetA-fwd and the reverse primer PstI_egfp_rev. | this work |  |
| ATG1-egfp-rev | TGCTCACATCCATTGTTCCCTC | pAUG1-egfp construction. Reverse primer for amplfication of the *metA* region from pJET-metA-a::b containing the Δ13-58 deletion. The primer contains the first 2 codons of *metA* (ATG1 was considered as the first codon) fused to third codon of *egfp*. Used with the forward primer NheI-PmetA-fwd. The amplicon was then used in an overlapping PCR with the forward primer NheI-PmetA-fwd and the reverse primer PstI_egfp_rev. | this work |  |
| TSS-ATG2-fwd | GGTGATGAGTGGACGCCCATG | pAUG2-egfp construction. Forward primer for inverse PCR of pJET-CD33 to obtain *metA* Δ13-124 deletion (+1 is the TSS), resulting in pJET-metA-a::c | this work |  |
| TSS-ATG2-rev | CCACTCATCACCACGGATCATT | pAUG2-egfp construction. Reverse primer for inverse PCR of pJET-CD33 to obtain *metA* Δ13-124 deletion (+1 is the TSS), resulting in pJET-metA-a::c | this work |  |
| ATG2-egfp-fwd | GCCCGTGAGCAAGGGCGAGG | pAUG2-egfp construction. Forward primer for *egfp* amplfication to clone a *metA'::egfp* fusion; contains ATG2 and the following codon fused to the third codon of *egfp*. Used with the reverse primer PstI_egfp_rev. The amplicon was then used in an overlapping PCR with the forward primer NheI-PmetA-fwd and the reverse primer PstI_egfp_rev. | this work |  |
| ATG2-egfp-rev | CTCACGGGCATGGGCGTCCA | pAUG2-egfp construction. Reverse primer for amplfication of the *metA* region from pJET-metA-a::c containing the Δ13-124 deletion. The primer contains ATG2 and the following codon fused to third codon of *egfp*. Used with the forward primer NheI-PmetA-fwd. The amplicon was then used in an overlapping PCR with the forward primer NheI-PmetA-fwd and the reverse primer PstI_egfp_rev. | this work |  |
| PstI_egfp_rev | AACTGCAGTTACTTGTACAGCTCGTCCATG | Reverse primer for amplification of *egfp* for cloning into pRS-T or pP_sinI_-T | this work |  |
| BamHI-egfp(CD3)-fwd | CGGGATCCAGCAAGGGCGAGGAGCTG | pRS-'egfp construction. Forward primer for amplifaction of *egfp* starting from the third codon; contains a BamHI restriction site. Used with the reverse primer PstI_egfp_rev | this work |  |
| BamHI-metA-CD3-rev | CGGGATCCGAAATCCATTGTTCCCTCGGC | pCD3-egfp construction. Reverse primer containing a BamHI restriction site, used with the forward primer NheI-PmetA-fwd | this work |  |
| BamHI-metA-CD16-rev | CGGGATCCCAAAAAAACCGGCGGCGA | pCD16-egfp construction. Reverse primer containing a BamHI restriction site, used with the forward primer NheI-PmetA-fwd | this work |  |
| BamHI-metA-CD33-rev | GCGGATCCGGGCAGCGTATCGGGTATCTTG | pCD33-egfp construction. Reverse primer containing a BamHI restriction site, used with the forward primer NheI-PmetA-fwd | this work |  |
| metA-ATG1m-fw | GGGAACATAGGATTTCCGAGGACCGGTC | pCD33-AUG1m-egfp construction. Forward primer for site directed mutagenesis AT68,69TA | this work |  |
| metA-ATG1m-rev | GGAAATCCTATGTTCCCTCGGCCCTTTAG | pCD33-AUG1m-egfp construction. Reverse primer for site directed mutagenesis AT68,69TA | this work |  |
| metA-ATG2m-fw | ACGCCCCTCCCCATCAAGATACCCGAT | pCD33-AUG2m-egfp construction. Forward primer for site directed mutagenesis A137C, G139C | this work |  |
| metA-ATG2m-rev | GATGGGGAGGGGCGTCCACTCGATC | pCD33-AUG2m-egfp construction. Reverse primer for site directed mutagenesis A137C, G139C | this work |  |
| metA-SDm-fwd | GCCGATTGAACAATGGATTTCCGAG | pCD33-SDm-egfp construction. Forward primer for site directed mutagenesis GG61,62TT | this work |  |
| metA-SDm-rev | TTGTTCAATCGGCCCTTTAGCGACTT | pCD33-SDm-egfp construction. Reverse primer for site directed mutagenesis GG61,62TT | this work |  |
| metA-SBPm-fwd | TCGCTGGGGGGCCGAGGGAACAAT | pCD33-SBPm-egfp construction. Forward primer for site directed mutagenesis AAA51,52,53TTT | this work |  |
| metA-SBPm-rev | TCGGCCCCCCAGCGACTTCTTTAAC | pCD33-SBPm-egfp construction. Reverse primer for site directed mutagenesis AAA51,52,53TTT | this work |  |
| metA-M1-fwd | CGCCGCCCCTTTTTTTGTATTCGATCGA | pCD33-M1-egfp construction Forward primer for site directed mutagenesis GG106,107CC destabilising the transcriptional terminator | this work |  |
| metA-M1-rev | AAAAAAAGGGGCGGCGAAACGATCGC | pCD33-M1-egfp construction. Reverse primer for site directed mutagenesis GG106,107CC destabilising the transcriptional terminator | this work |  |
| HindIII-Bj-Trrn-Fwd | CAGAAGCTTAGGCCTGCCAAGGACAAG | pSRKGm-T construction. Forward primer for *B. japonicum* *rrn* sequence amplification for cloning using HindIII into pSRKGm. | this work |  |
| HindIII-Bj-Trrn-rev | CCCAAGCTTCAATTCTTGAAGACCACGGC | pSRKGm-T construction. Reverse primer for *B. japonicum* *rrn* sequence amplification for cloning using HindIII into pSRKGm. | this work |  |
| EcoRI_del_RA2_up_Fw | AAAGAATTCGCGTTTCGGGGATAAGGCAG | pK18mobsacB-ΔRA construction. Forward primer for amplification of the upstream region, which flanks the sequence that should be deleted | this work |  |
| Del_RA2_up_RV | GCCGTCGACGGATCCGAGGCAATCATTTGATAGCGCGAGGAGG | pK18mobsacB-ΔRA construction. Reverse primer for amplification of the upstream region, which flanks the sequence that should be deleted | this work |  |
| Del_RA2_dw_Fw | TGCCTCGGATCCGTCGACGGCCGAGGGAACAATGGATTTCCGAGG | pK18mobsacB-ΔRA construction. Forward primer for amplification of the downstream region, which flanks the sequence that should be deleted | this work |  |
| XbaI_del_RA2_dw_Rv | GCTCTAGACTCGGTCTTGATCTTGTTGGGC | pK18mobsacB-ΔRA construction. Reverse primer for amplification of thedownstream region, which flanks the sequence that should be deleted | this work |  |
| Contr_del_RA2 Fw | CCTCCTCGCGCTATCAAATGAT | Test-PCR to distinguish strains *S. meliloti* 2011 and 2011 ΔRA*,* forward primer | this work |  |
| Contr_del_RA2_Rv | CCGGTCCTCGGAAATCCAT | Test-PCR to distinguish strains *S. meliloti* 2011 and 2011 ΔRA*,* reverse primer | this work |  |
| pSRK-rev | CAGCAATAGACATAAGCGG | Sequencing primer for pSRKGm | this work |  |
| pRS1-rev | GTTATTGGTGCCCTTAAACGC | Sequencing primer for pRS1 | this work |  |

*Primer pair efficiency is given for primer pairs used in qPCR analysis.
