# Supplementary material for: Incoherent dual regulation by a SAM-II riboswitch controlling translation at a distance
Source: RNA Biol. 2022 Aug 11;19(1):980–95. doi: 10.1080/15476286.2022.2110380 (PMC9373788; doi:10.1080/15476286.2022.2110380)
Supplement: Supplemental Material [file KRNB_A_2110380_SM3921.zip › Suppl_Figs_SAM_RNA_Biol_R1_final.pdf]

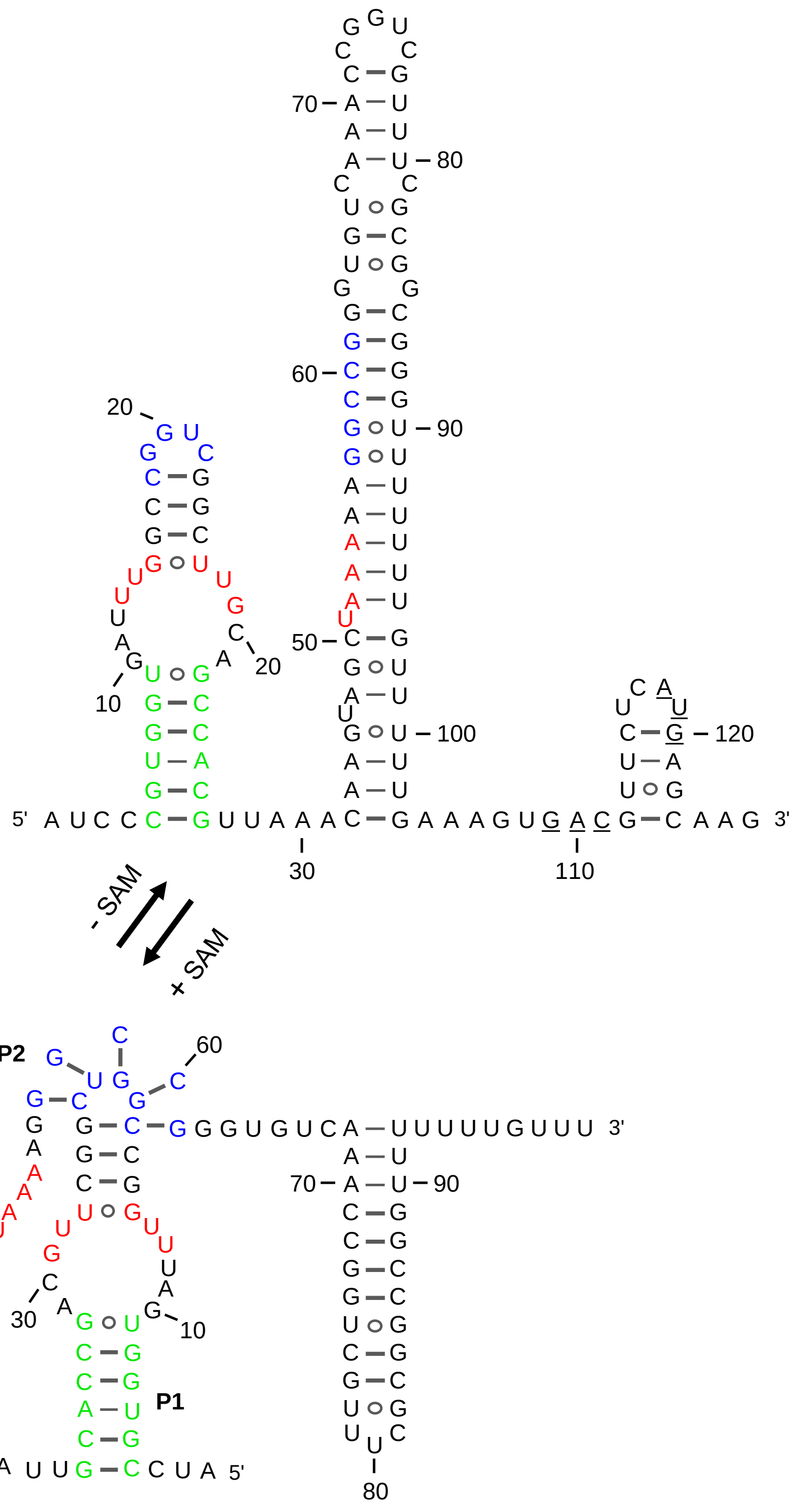

Figure S1 The *metZ* riboswitch and downstream sequences. Shown are the predicted alternative RNA structures that are adopted without SAM binding (-SAM) and upon interaction with SAM (+ SAM). Red nucleotides: the SAM-binding pocket (SBP); green nucleotides: sequences building stem 1 (P1) upon SAM binding; dark blue nucleotides: sequences building a pseudoknot (P2) upon SAM binding (according to Corbino et al., 2005). Upon SAM binding, a transcriptional terminator structure is formed. The translational start codon of *metZ* is located downstream of the terminator (not shown).

Fig. S2

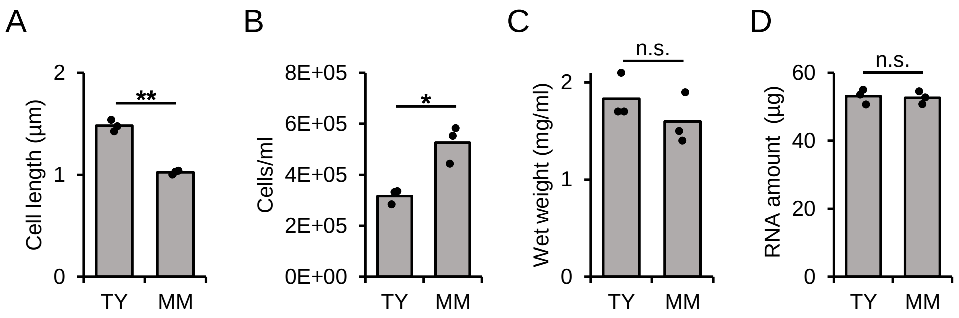

Figure S2 Comparison of *S. meliloti* 2011 cultures grown in TY and MM to an OD_{600} of 0.5. A) Cell length (n = 350). **B)** Cell count per ml. **C)** Wet weight of the cell pellet of 1 ml culture. **D)** RNA amount isolated from cells of 15 ml culture. All graphs show means and single data points of three independent experiments.

Fig. S3

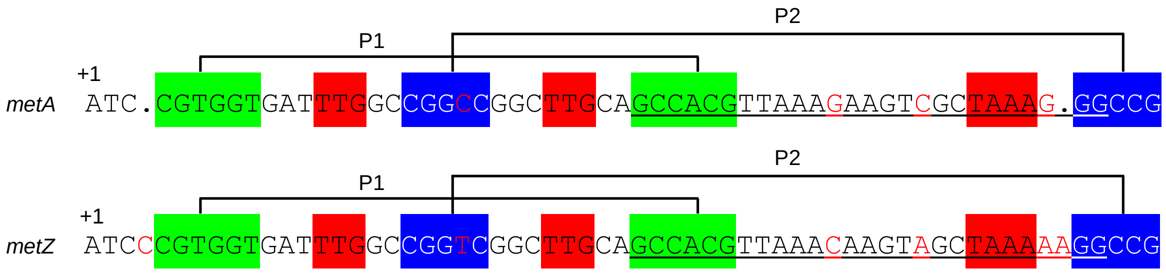

Figure S3 The *metA* and *metZ* aptamers (DNA sequence is shown) exhibit high sequence similarity, but allow for specific detection of the riboswitch-containing sRNAs in Northern blots. Nucleotides corresponding to the SAM-binding pocket (red), the P1 stem-loop (green) and the P2 pseudoknot (blue) are highlighted (see also Fig. S1). Differences in the sequences are indicated by red letters. Regions targeted by the Northern hybridization probes are underlined.

Fig. S4

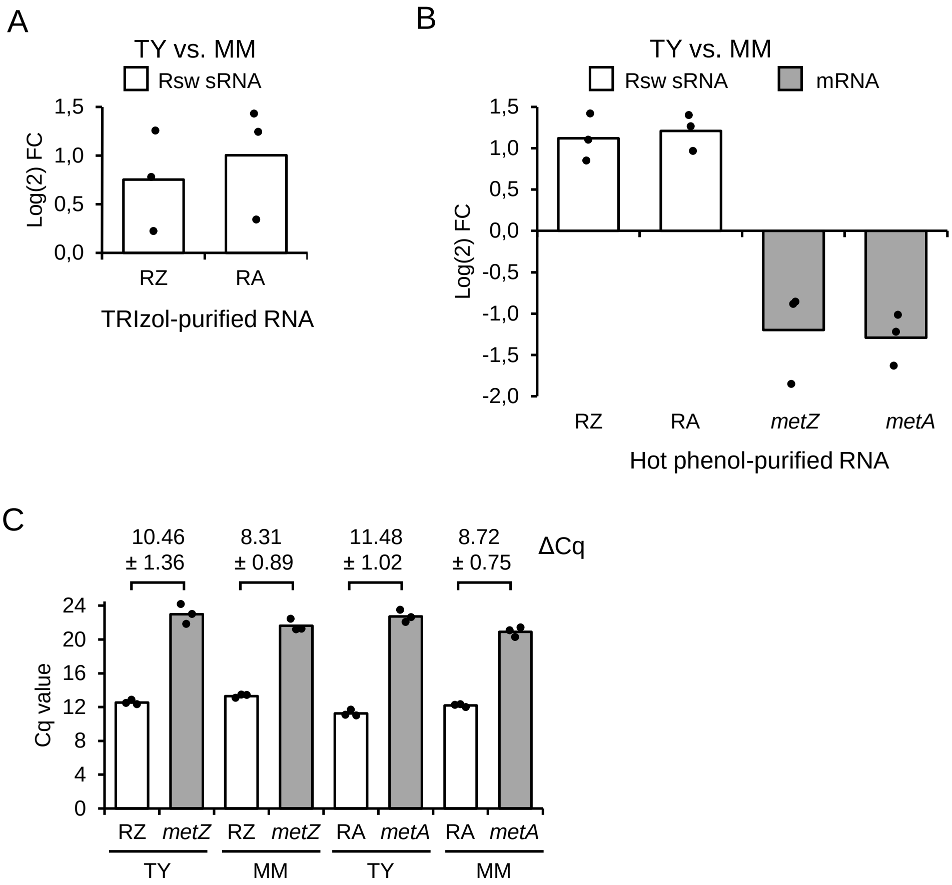

Figure S4 Analysis of RNA steady state amounts, in *S. meliloti* 2011 cultures grown in TY and MM, using RNA purified with different methods. A) and **B)** Comparison of RNA levels by qRT-PCR analysis of TRIzol-purified or hot-phenol-purified RNA. Primers depicted in Fig. 1A were used. The sRNA-detecting primers bind also to the 5'-UTR of the respective mRNA (RA: RA1 + RA2). However, the sRNAs are present in much higher amounts than the mRNAs (Fig. 2D and panel C), and therefore using the sRNA-detecting primers, changes in the sRNA levels could be revealed. The data show that both RNA purification methods lead to similar results when differences in the RNA levels in cultures grown in TY or MM are studied by qRT-PCR (for sRNA, compare panels A and B; for mRNA, compare panel B to Fig. 2C). Additionally, both qRT-PCR and Northern blot reveal higher sRNA levels in MM than in TY, when TRIzol-purified RNA is used (compare panel A to Fig. 2C). **C)** Cq values of the qRT-PCR analysis conducted with TRIzol-purified RNA and the sRNA-detecting or the mRNA-specific primers (see Fig. 1A). In the TRIzol-purified RNA, sRNAs are enriched and long RNAs are isolated with lower efficiency. This explains the differences in the ΔCq values when compared to the analysis of hot phenol-purified RNA using the same primers (see Fig. 2D). All graphs show means and single data points of three independent experiments.

Fig. S5

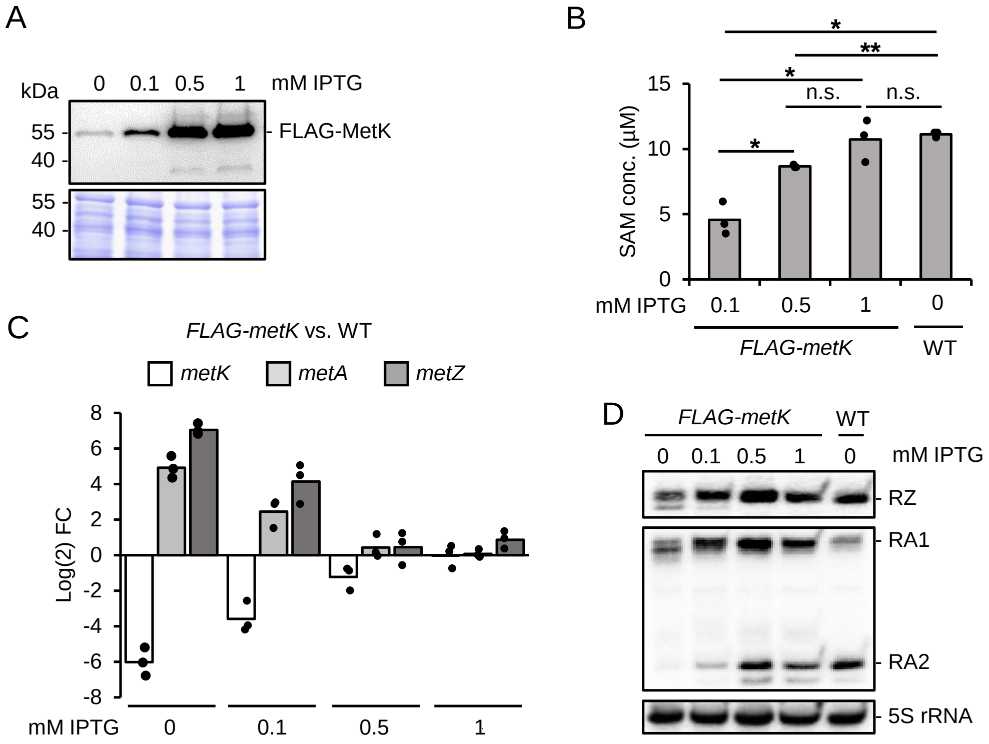

Figure S5 Manipulation of SAM and RNA levels by IPTG induction of the SAM synthetase gene in the mutant 2011 *FLAG-metK*. Pre-cultures were grown with 1 mM IPTG in TY medium. The next day the culture was diluted to $OD_{600\ nm}$ of 0.2 and crystal violet was added to induce the production of the LacI repressor. The diluted culture was divided in four portions and IPTG was omitted or added at the indicated concentrations to induce expression of *FLAG-metK*. Cells were harvested at the $OD_{600\ nm}$ of 0.5 and used for protein and RNA analysis. **A)** Western blot analysis showing an increase in the FLAG-MetK amount at increasing IPTG concentrations. Migration of protein marker bands (in kDa) is indicated. **B)** SAM amount in the cultures shown in A) and in two additional, independent cultures. **C)** Comparison of the levels of the indicated mRNAs, as determined by qRT-PCR and shown as $\log_2$FC, in the 2011 *FLAG-metK* mutant and the parental strain 2011 (WT). **D)** Northern blot hybridization of total RNA separated in denaturing 10 % PAA gel with probes detecting the indicated sRNAs. Used strains and IPTG concentrations are indicated. The graphs show means and single data points of three independent experiments. Each qRT-PCR experiment was performed in technical duplicates.

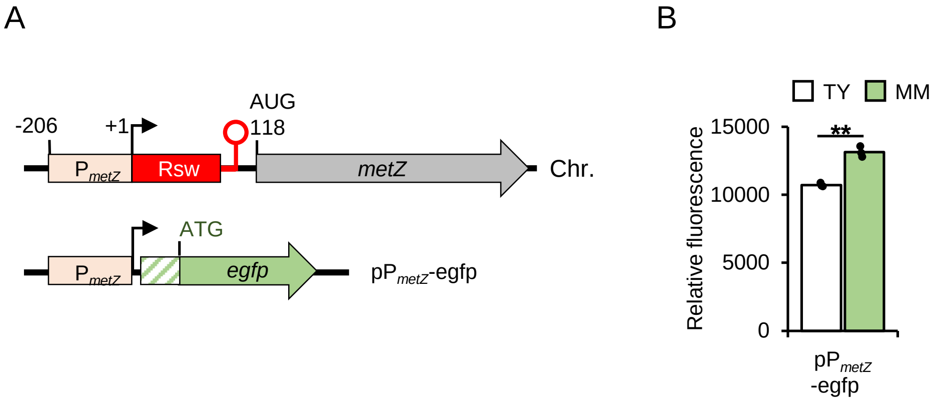

Figure S6 Higher *metZ* promoter activity in MM than in TY medium. A) Schematic representation of *metZ* on the chromosome (Chr.) and of plasmid pP_{metZ}-egfp carrying a promoter fusion with *egfp* (green arrow). The hatched box represent a synthetic 5'-UTR containing a typical Shine-Dalgarno sequence. Additional elements corresponding to the *metZ* transcript are indicated. For other information see Fig. 1A. **B)** Relative fluorescence of TY and MM cultures of *S. meliloti* 2011 carrying pP_{metZ}-egfp. The graph shows means of three independent experiments and single measure points representing means from three technical replicates of each independent experiment.

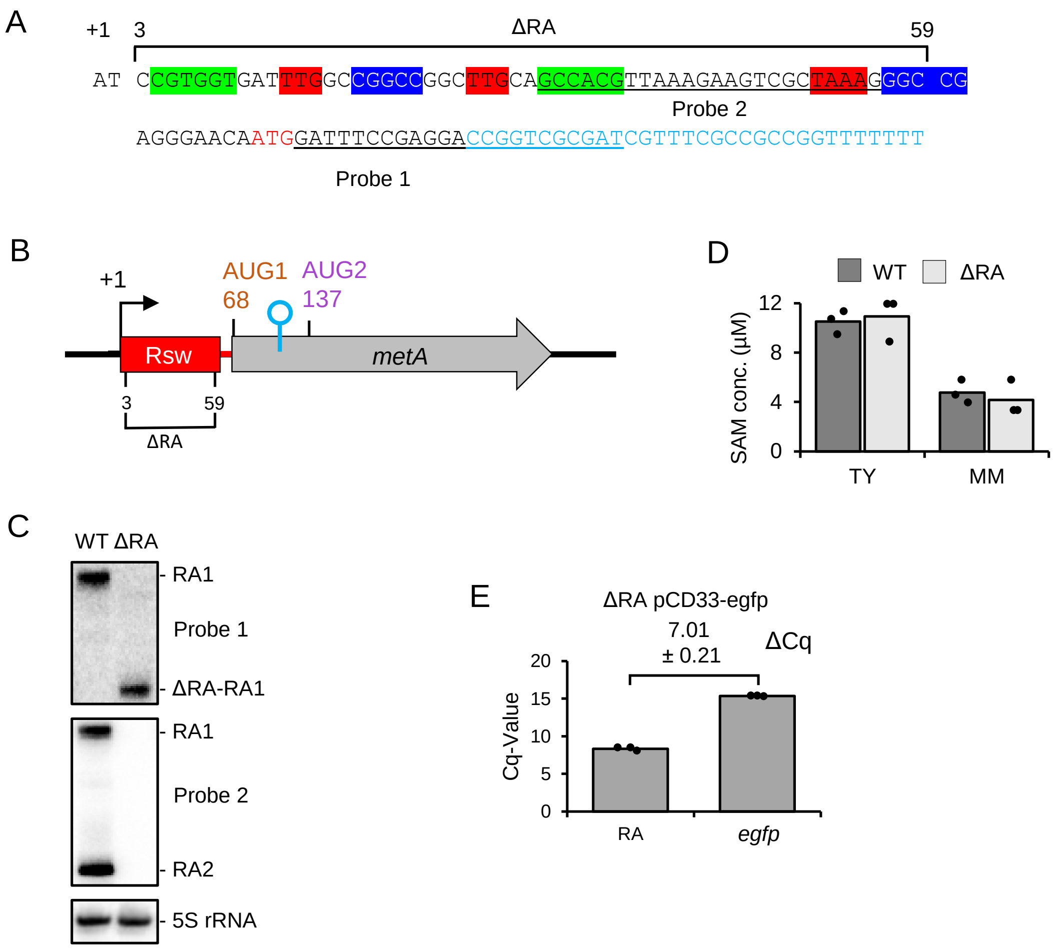

Figure S7 Characterization of the *S. meliloti* 2011 ΔRA mutant. A) RA1 sequence with indicated ΔRA deletion in the aptamer region (compare to Fig. S3 and Fig. 1B). Regions targeted by probes used for Northern blot hybridization are underlined. +1 indicates the transcription start site (TSS). **B)** Scheme of the *metA* locus with the indicated aptamer deletion, showing that in the mutant strain the TSS and the terminator restricting *metA* expression are preserved. **C)** Northern blot hybridization with the indicated probes (see panel A) confirming the aptamer deletion. **D)** SAM levels in the parental strain (WT) and the ΔRA mutant grown in the indicated media. **E)** Cq values of the qRT-PCR analysis of *S. meliloti* 2011 ΔRA pCD33-egfp cultures grown in TY, using primers directed against the *egfp* or the riboswitch part of the fusion mRNA CD33-egfp (see Fig. 7A; RA: RA1 + RA2). Hot phenol-purified total RNA was used. The difference in the Cq values (mean and s. d.) is given above the bars. The difference of 7 cycles indicates much higher abundance of the sRNAs in comparison to the mRNAs. All graphs show means and single data points of three independent experiments.
